# Resorbable Nanomatrices from Microbial Polyhydroxyalkanoates: Design Strategy and Characterization

**DOI:** 10.3390/nano12213843

**Published:** 2022-10-31

**Authors:** Ekaterina I. Shishatskaya, Alexey E. Dudaev, Tatiana G. Volova

**Affiliations:** 1 Department of Medical Biology, School of Fundamental Biology and Biotechnology, Siberian Federal University, 79 Svobodnyi Av., 660041 Krasnoyarsk, Russia; 2 Chemistry Engineering Centre, ITMO University, Kronverkskiy Prospekt, 49A, 197101 Saint Petersburg, Russia; 3 Institute of Biophysics SB RAS, Federal Research Center “Krasnoyarsk Science Center SB RAS”, 50/50 Akademgorodok, 660036 Krasnoyarsk, Russia; 4 Basic Department of Biotechnology, School of Fundamental Biology and Biotechnology, Siberian Federal University, 79 Svobodnyi Av., 660041 Krasnoyarsk, Russia

**Keywords:** biodegradable polyhydroxyalkanoates (PHAs), copolymers, films, nanomembranes, properties, cell cultures, wound coverings, healing of skin wounds

## Abstract

From a series of biodegradable natural polymers of polyhydroxyalkanoates (PHAs)—poly-3-hydroxybutyrate (P(3HB) and copolymers containing, in addition to 3HB monomers, monomers of 3-hydroxyvalerate (3HV), 3-hydroxyhexanoate (3HHx), and 4-hydroxybutyrate (4HB), with different ratios of monomers poured—solvent casting films and nanomembranes with oriented and non-oriented ultrathin fibers were obtained by electrostatic molding. With the use of SEM, AFM, and measurement of contact angles and energy characteristics, the surface properties and mechanical and biological properties of the polymer products were studied depending on the method of production and the composition of PHAs. It has been shown in cultures of mouse fibroblasts of the NIH 3T3 line and diploid human embryonic cells of the M22 line that elastic films and nanomembranes composed of P(3HB-co-4HB) copolymers have high biocompatibility and provide adhesion, proliferation and preservation of the high physiological activity of cells for up to 7 days. Polymer films, namely oriented and non-oriented nanomembranes coated with type 1 collagen, are positively evaluated as experimental wound dressings in experiments on laboratory animals with model and surgical skin lesions. The results of planimetric measurements of the dynamics of wound healing and analysis of histological sections showed the regeneration of model skin defects in groups of animals using experimental wound dressings from P(3HB-co-4HB) of all types, but most actively when using non-oriented nanomembranes obtained by electrospinning. The study highlights the importance of nonwoven nanomembranes obtained by electrospinning from degradable low-crystalline copolymers P(3HB-co-4HB) in the effectiveness of the skin wound healing process.

## 1. Introduction

Polymeric nanostructured materials (NSPs), characterized by different morphologies and differences in spatial organization, play an increasingly significant role in various fields. Such materials are promising for a wide range of biological and technological applications, primarily for medicine and pharmaceuticals, causing a revolutionary transformation in existing diagnostic and treatment technologies [1]. Nanomaterials provide a significant improvement in the quality of care by increasing the accuracy and reliability of diagnostics, with more efficient targeting of therapeutic agents while minimizing side effects, as well as increasing the effectiveness of the latest technologies in regenerative medicine [2,3]. Nanomaterials with a high surface to volume ratio demonstrate unique physicochemical properties and are promising not only for drug and gene delivery but also as biosensors, in cell and tissue engineering technologies, etc. [4]. The rapid and growing interest in NSPs is related to their properties. Size, shape, composition, molecular engineering, assembly, and nanostructures are the key parameters that characterize NSPs, govern their functions, and allow them to be applied in various fields. Polymeric nanostructured materials include various types of nanostructures, such as micelles, polymersomes, nanoparticles, nanocapsules, nanogels, nanofibers, dendrimers, brush polymers, and nanocomposites [5]. Their properties, such as stability, size, shape, surface charge, surface chemistry, mechanical strength, porosity, etc., can be tailored to specific functions that are matched to meet the needs of the targeted biomedical application [2]. Such nanostructured materials can be obtained by various methods, including direct dissolution, film casting and dialysis methods, electrospinning, micronization of oil-in-water emulsions, and others [6,7,8,9]. In general, polymeric nanostructured materials should have properties appropriate for specific biomedical applications.

One of the most promising areas of application of new-generation nanomaterials is reconstructive medicine based on cell biology and tissue engineering technologies [9,10]. To develop and improve the methods of reconstructive medicine based on tissue engineering, new materials of high functionality and specificity are required, including the design of systems capable of reproducing the biological functions of a living organism. The main focus of researchers today is the search for technologies to create bioartificial materials and organs, which are a system of materials of artificial or biological origin, including functioning cells, or stimulating the regeneration of the corresponding cells in the implantation zone [11,12]. Scaffolds should have multifunctionality, sufficient mechanical strength and elasticity, biocompatibility at the protein and cellular level, the ability to attach to cells and stimulate cell proliferation and differentiation, the capacity for neovascularization, and the possibility of sterilization without changing the medical and technical properties [13,14]. The use of such bioconstructions, additionally loaded with drugs, is a revolutionary trend in reconstructive surgery and transplantation and has great prospects. Matrices of this type include membranes, films, and grids; this is the simplest type of open system [15]. The flat surface of the films appears to be an acceptable substrate for culturing in vitro. The advantages of such systems lie in the relative ease of manufacture and use. Thus, porous mesh matrices are obtained by various methods; these are fiber weaving, solution deep molding, leaching of salt particles, gas foaming, freeze drying, and extrusion [16,17].

The use of the potential of cellular technologies for reconstructive purposes is implemented using several approaches. In one of them, a suspension or cluster of cells of the required phenotype grown in vitro is introduced into damaged organ tissues directly or into the bloodstream [18]. In another, technologically more complex approach, cells are grown outside the body on a matrix (scaffold), and then the bioengineered structure or the formed tissue is implanted into the recipient organism. The success of the second method depends largely on the properties of the scaffolds used as cell carriers [11,19]. All the necessary properties of the matrix are determined by the properties of the source material and the technology of its processing. Therefore, the key problem for the success of creating effective bioconstructs is the availability of an adequate biodegradable and biocompatible material.

The main requirement for biomaterials for biomedical purposes is biocompatibility [20,21]. The factors that determine biocompatibility include the composition of the material, the structure and properties of the surfaces of the matrices, the shape of the cell carrier, and, at the organismal level, the site of implantation and the age of the organism, as well as the degree of tissue damage [22,23]. One of the indicators of biocompatibility is the ability of the matrix surface to maintain cell adhesion, which further affects cell proliferation and the success of the “cell–matrix” structure [24]. The factors influencing the attachment of cells to matrices include the hydrophilicity of the surface. Cell adhesion is favorable on scaffold surfaces with medium hydrophobicity, with contact angles up to 70° [25,26]. To increase the hydrophilicity of the surface of the material, various methods are used—treatment with γ-radiation, or with plasma of oxygen, hydrogen, or nitrogen [27,28,29]—which increase the wettability due to the formation of polar groups on the surface, as well as grafting hydrophilic molecules to the surface of the matrices, which can carry an additional functional load (hyaluronic acid, carboxylic acids, chitosan), which gives the product antibacterial activity [30,31].

An important parameter of cell carriers, surface roughness is a parameter that, at the nanolevel, affects the adsorption of proteins, cell attachment, and their further growth and development. For different types of cells, surfaces with different roughness coefficients are more suitable. For example, a rougher surface with a unidirectional surface pattern structure is better suited for osteoblasts [32]; the adhesion, proliferation, and differentiation of bone marrow cells increase with increasing matrix surface roughness [33], while a smoother surface is suitable for epithelial cells. In addition, roughness determines the hydrophilicity of the surface; as was shown by De Gennes, the presence of small differences in the surface relief leads to an increase in the energy barrier of the surface and a decrease in the wetting angle and improves the wettability of the surface, and vice versa—large differences in the topographic structure lead to an increase in hydrophobicity [34].

The most important requirement for biomaterials used in reconstructive technologies is the ability of the materials to degrade in the body, without the formation of toxic products and at a rate comparable to the rate of tissue regeneration [35]. The permanent presence of an implant in the body is associated with undesirable side effects: surface oxidation, inflammatory reactions, shielding of the mechanical load necessary for normal tissue development, and the need for a second operation to remove the implant [36]. Today, biodegradable implants are most in demand; they are destroyed in the body at the same time as they are replaced by healthy organs or tissues.

Among degradable polymeric materials, an important position is occupied by polyhydroxyalkanoates (PHAs)—polymers of microbiological origin. PHAs are a family of biodegradable thermoplastic polymers with different chemical structures and different physicochemical properties [37,38,39,40,41,42,43,44]. These polymers can be processed into products using known and accessible methods from various phase states (solutions, emulsions, powders, melts) [45,46], as well as being used to obtain composites with various fillers and materials [47]. PHAs’ prospects for application include various fields, from municipal and agricultural to medicine and pharmacology [48,49,50,51], and they have great potential to contribute to the “circular economy” [52]. The high biological compatibility of PHAs at the cellular, tissue, and organism levels, and their long-term and controlled resorption in vivo, makes PHAs some of the most promising materials for biomedical applications. A particularly promising field of application of PHAs is reconstructive medicine, based on cell and tissue engineering technologies [6,53,54,55,56,57]. An important aspect of the prospects of PHAs is the possibility of synthesizing polymers of various chemical compositions and obtaining polymer products with different mechanical strength indices, as well as surface structures and properties.

The most common and the best-studied PHA is the homopolymer of 3-hydroxybutyric acid (poly-3-hydroxybutyrate, P(3HB)). Despite the great potential of this polymer, its high crystallinity (above 70%) and hydrophobicity limit its use. P(3HB) does not crystallize to form an ordered structure, and it is difficult to process P(3HB) into products, which demonstrate low shock resistance and rigidity and are prone to “physical ageing” [12,58]. Properties of polymeric materials, including P(3HB), can be improved by using biological, chemical, and physical methods, such as the fabrication of P(3HB) composites with other materials, biosynthesis of PHA copolymers, chemical modification, and physical treatment of the surfaces of polymer products [47,59,60,61,62,63,64,65]. These methods help to change the properties of polymer products, increase their biodegradation rate, enhance their flexibility and mechanical strength, increase the surface hydrophilicity and porosity to facilitate cell attachment, improve the gas dynamic properties of the products, and enhance their permeability to the substrates and metabolic products of cells and tissues.

The present work shows the possibilities of constructing cellular nanomatrices from PHAs, which differ in microstructure and surface properties, and the potential of using various methods for processing these biopolymers in order to modify the characteristics and use of the resulting polymer nanoproducts.

## 2. Materials and Methods

### 2.1. Production of PHAs

A family of PHAs with different sets and ratios of monomers has been investigated (Table 1): the homopolymer of 3-hydroxybutyrate P(3HB) [-O-CH(CH_3_)-CH_2_-CO-] and copolymers, each consisting of the 3-hydroxybutyrate monomer and another monomer. Secondary monomers differed in their structure and carbon chain length: 4-hydroxybutyrate (4HB) [-O-CH_2_-CH_2_-CH_2_-CO-], 3-hydroxyvalerate (3HV) [-CH(C_2_H_5_)-CH_2_-CO-], and 3-hydroxyhexanoate (3HHx) [-O-CH(C_3_H_7_)-CH_2_-CO-]. PHAs were synthesized using the *Cupriavidus necator* B-10646 bacterial strain and proprietary technology. The method of polymer synthesis, the composition and properties, and the research methods were described in detail previously [66]. The investigated copolymer samples contained macroinclusions of the second monomers, approximately 10 and 30 mol.%.

The polymer was extracted from the cell biomass with dichloromethane; the resulting extract was concentrated on an R/210V rotary evaporator (Büchi, Flawil, Switzerland) and then precipitated with ethanol. Repeating the procedures of polymer dissolution and reprecipitation ensured the removal of impurities and allowed homogeneous samples to be obtained. The polymer samples were dried in a fume hood at room temperature for 72 h. The purity of the polymer and copolymers was determined by chromatography of methyl esters of fatty acids after methanolysis of purified polymer samples using a 7890A chromatograph–mass spectrometer (Agilent Technologies, Santa Clara, CA, USA) equipped with a 5975C mass detector (Agilent Technologies, U.S [67]).

### 2.2. Production of Polymer Films

PHA films were prepared by casting a 2% polymer solution in dichloromethane in degreased Teflon-coated molds, and then the films were left in a laminar flow cabinet (Labconco, Kansas City, MO, USA) for 72 h and further dried until complete evaporation of the solvent in a vacuum dissipator (Labconco U.S.) or in a thermostat at 40 °C (evaporation boiling temperature of dichloromethane).

### 2.3. Production of PHA Nanomembranes by Electrostatic Spinning

Ultrafine fiber nanomembranes were produced by electrospinning from PHA solutions using a Nanon 01A automatic set-up (MECC Inc., Ogori, Japan). Chloroform solutions with polymer concentrations varying from 1 to 10 wt.% were prepared from all types of PHAs. The polymer solution was poured into a plastic syringe (13 mm inside diameter). The syringe was fixed horizontally in the set-up; the solution feeding rate was varied from 4 to 8 mL/h, the applied voltage from 15 to 30 kV, and the working distance from 11 to 15 cm. Randomly oriented or aligned ultrafine fibers were collected on a flat steel plate or a rotating drum (at 1000 rpm), respectively; both collectors were covered with aluminum foil to collect ultrafine fibers more effectively.

### 2.4. A Study of PHA Films’ and Nanomembranes’ Properties

The surface microstructure of PHA films was analyzed using scanning electron microscopy (FE-SEM S-5500 high-resolution scanning electron microscope, Hitachi, Tokyo, Japan). Prior to microscopy, the samples were sputter-coated with platinum (at 25 mA, for 60 s), using an EM ACE200 (“Leica”, Vienna, Austria). Surface properties were studied with a Drop Shape Analyzer, DSA-25E (Krüss, Germany), using the DSA-4 software for Windows. Surface properties such as surface free energy (γS), interfacial free energy (γSL), and cohesive forces (WSL, erg/cm^2^) were calculated based on the measured water contact angles (θ, °), using the de Gennes equations [34]. Porosity, including the number, size, and total area occupied by pores, was analyzed from SEM images of the samples using Image J v1.53k.

The roughness of the film surface was determined using atomic-force microscopy (AFM) in semicontact mode (DPN 5000, NanoInk, Skokie, IL, USA). The arithmetic mean surface roughness (Sa) and the root mean square roughness (Sq) were determined based on 10 points, as the arithmetic averages of the absolute values of the vertical deviations of the five highest peaks and lowest valleys from the mean line of the surface profile, using conventional equations [68]. AFM data were processed and statistical analysis of the images was performed using the open source Gwyddion (2.51) free software for image analysis and processing.

The physical and mechanical characteristics of the samples were recorded using an Instron 5565 universal tensile testing machine (Great Britain) with the measurement of the Young’s modulus (E, MPa), absolute tensile strength (σ, MPa), and elongation at break (ε, %).

### 2.5. A Study of the Biological Compatibility of PHA Films and Nanomembranes in Cell Cultures In Vitro

PHA samples loaded with drugs were shaped as 10 mm diameter disks. They were placed into 24-well culture plates (TPP Techno Plastic Products AG, Trasadingen, Switzerland) and sterilized in a Sterrad NX medical sterilizer (Johnson & Johnson, New Brunswick, NJ, USA). Scaffolds were seeded with cells at 105 cells per scaffold for 2 to 11 days. Mouse fibroblast NIH 3T3 cells, and a culture of diploid human embryonic cells—fibroblasts of the M22 line—were cultured in Dulbecco’s Modified Eagle’s Medium (DMEM, Gibco, Grand Island, NY, USA) supplemented with 10% fetal bovine serum (FBS, HyClone, Logan, UT, USA) and 1% antibiotic–antimycotic solution (Sigma-Aldrich, St. Louis, MO, USA) in a 5% CO_2_ atmosphere at 37 °C. Cell morphology (shape, state of the cytoplasmic membrane, presence of vacuolization, granularity, inclusions) and cultural properties (adhesion, number of cells, proliferation index) were analyzed. The proliferation index (IP3) was determined as the ratio of the number of cells in a well on the 3rd day of cultivation to the number of cells on the 1st day of cultivation. Viability of cultured fibroblasts was evaluated using the 3-(4,5-dimethylthiazol-2-yl)-2,5-diphenyl tetrazolium bromide (MTT test) (Sigma, Ronkonkoma, NY, USA) assay. The optical density of the samples was measured at wavelength 540 nm, using an iMark microplate reader (Bio-Rad LABORATORIES Inc., Hercules, CA, USA). The number of viable cells was determined from the calibration graph.

### 2.6. An In Vivo Study of P(3HB-co-4HB) Films and Nanomembranes as Experimental Wound Dressings

Experiments were conducted in accordance with the Russian State Standard [69] and international regulations [70,71,72]. The protocol of the experiments was approved by the Local Ethics Committee at the Siberian Federal University. The experiment was performed on female Balb/c mice weighing 20–22 g. Under inhalation ether anesthesia under aseptic conditions, one piece of skin with an area of approximately 1 cm^2^ was excised on the back of each animal, after shearing the hair. The animals were divided into 4 groups, 6 mice per group. In the treatment groups, three types of experimental wound dressings were used: P(3HB-co-4HB) type 1 collagen-coated products, solvent casting films, and random and aligned nanomembranes, which were covered with an aseptic dressing. In the control group, the wound defect was closed with an aseptic gauze bandage. Bandages were replaced with fresh ones daily. The healing process was assessed planimetrically via the area and speed of wound healing and via the results of histological studies. After sacrificing the animals using an overdose of ether anesthesia, the material was taken for histological analysis. Transverse histological sections of skin wounds with a thickness of 7 μm were examined on the 10th day. Sections were stained with hematoxylin and eosin.

### 2.7. Statistical Analysis

Statistical analysis of the results was performed via conventional methods, using the standard software package of Microsoft Excel. Each experiment was performed in triplicate. Arithmetic means and standard deviations were obtained. The statistical significance of results was determined using Student’s *t* test (significance level: *p* ≤ 0.05).

## 3. Results and Discussion

The most accessible and mastered cell matrices include membranes, films, and nets. This is the simplest type of open system. The flat surface of the film appears to be an acceptable substrate for culturing cells in vitro. The advantages of such systems lie in the relative ease of manufacture and use. Film matrices are produced by a variety of methods, including fiber weaving, solution deep casting, salt particle leaching, gas foaming, freeze drying, extrusion, and more. The existence of PHA samples of different chemical composition made it possible to obtain film polymer systems with different surface microstructures and properties by various methods.

### 3.1. Properties of Solvent Casting PHA Films

The study of the effect of PHA composition on the characteristics of polymer-cast films included SEM and AFM, measurement of contact angles of wetting with liquids, and assessment of the energy characteristics of the surface. The microstructures and surface properties of polymer films obtained from PHA of various compositions, with significantly different basic physicochemical properties (crystallinity, molecular weight, and temperature characteristics), were studied (Table 1). 

**Figure 1:**
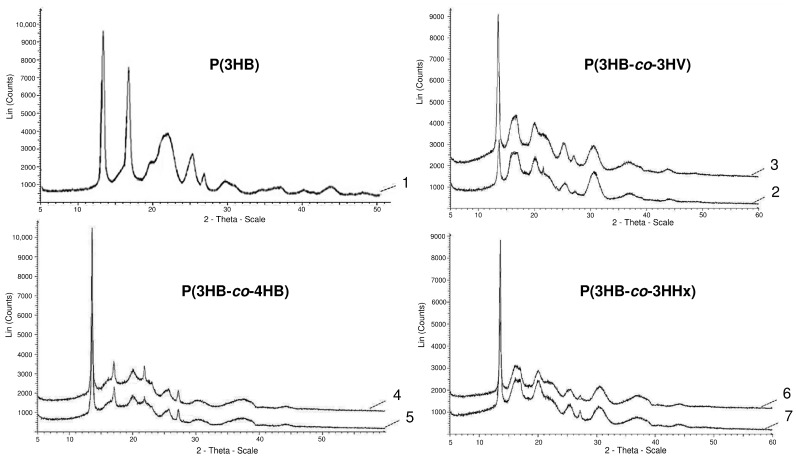
X-ray spectra of PHA samples with different sets of monomers (the numbering indicating the composition of the copolymer is similar to that in Table 1).

The most significant changes in the compositions of PHA monomers are reflected in the ratio of ordered and disordered phases, the indicator of which is the degree of crystallinity (C_x_). The lowest C_x_ values are typical for P(3HB-co-4HB) copolymers; this is confirmed by the obtained X-ray spectra (Figure 1).

**Figure 2:**
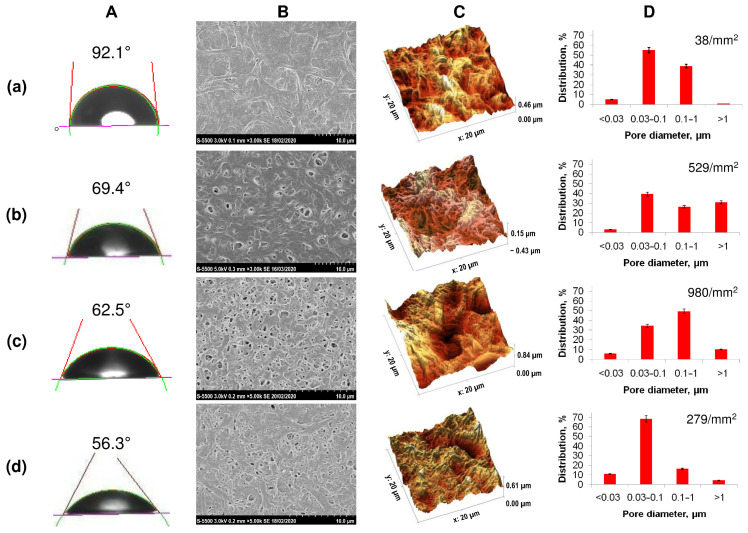
Solvent-cast PHA films’ characteristics: (**A**)—water contact angle, (**B**)—SEM image, (**C**)—roughness (AFM), (**D**)—porosity, where (**a**)—P(3HB), (**b**)—P(3HB-co-27 mol.% 3HV), (**c**)—P(3HB-co-35 mol.% 4HB), (**d**)—P(3HB-co-38 mol.% 3HHx).

The initial films with the same thickness (approximately 100 μm) had significant differences in morphology and surface characteristics. In contrast to homogeneous P(3HB), samples obtained from all types of copolymers had increased roughness values and reduced values of the contact angle of wetting with water (Figure 2, Table 2).

All copolymer samples had more pronounced porosity compared to films obtained from homogeneous P(3HB), in which the number of single pores was 38 pieces/mm^2^ with an average pore size of 0.5–0.7 µm. The presence of a large number of pores of various sizes, including larger pores with a diameter of 1.5 to 3.0 µm, was noted for film samples produced from the less crystalline P(3HB-co-4HB) and P(3HB-co-3HV) copolymers. P(3HB-co-3HHx) films are characterized by the presence of multiple, but smaller, pores ranging in size from 0.5 to 1.0–1.5 µm. Along with the size, the number of pores in films of different compositions also varied significantly, ranging from several pieces per unit area for films of (P(3HB) to several tens or more for copolymer films. The number of pores in films of P(3HB-co-3HV) was many times higher than those of homopolymer films and amounted to 529 pcs/mm^2^ with an average size of 0.085 µm. The porosity was somewhat lower for films composed of P(3HB-co-3HHx), 279 pieces/mm^2^, with an average size of 0.045 µm. The highest porosity is typical for P(3HB-co-4HB) films (980 pores/mm^2^) with an average size of 0.164 µm. Thus, PHAs of various chemical compositions make it possible to obtain cast films with various numbers and sizes of pores. This is apparently due to differences in the crystallization kinetics during film formation, as the solvent evaporates from polymer solutions with different C_x_ values, which can affect the attachment and development of eukaryotic cells. 

The hydrophilic/hydrophobic balance of a surface is a parameter that indirectly characterizes the hydrophilicity of a sample, influencing the adhesive properties. An indicator of this ratio is the value of the contact angle of the wetting of the surface with liquids. The films obtained from highly crystalline P(3HB) had the highest angle with respect to water (92.1°); the calculated values of the surface energy, the free energy of the interfacial surface, and the adhesion forces were 30.8, 8.92, and 94.74 erg/cm^2^. The value of the contact angle for all copolymer films was found to be reduced. This showed the hydrophilization of the surfaces of the copolymer samples (Table 2). At the same time, the decrease in the indicator was expressed in different ways. For films of copolymeric PHAs of various compositions, it was minimal (56.3°) for the copolymer with medium-chain 3-hexanoate and maximal (73.2°) for the P(3HB-co-4HB) sample, with some differences in the surface energy index (maximum value 57.1, minimum 41.1 mN/m).

The mechanical properties of solvent-cast films are relatively low. For all samples, regardless of the composition of the PHA used, the values of Young’s modulus and tensile strength were at the level of 1600 ± 500 nm and 12.50 ± 5.50 nm, respectively. The only exception was the value of elongation at break, which was several times (up to 450.4 ± 59.8%) higher than the values for samples from P(3HB-co-4HB) compared to other copolymers (213.9 ± 106.1%) and orders of magnitude higher than for homogeneous P(3HB) (3.1 ± 0.4%).

The results of the study of polymer films by atomic-force microscopy showed the effect of the chemical composition of the PHA on the surface roughness (Figure 2; Table 2). The lowest values of all studied parameters (Sa, Sq, Sz), namely 154, 180, 1255.9 nm, respectively, were recorded for the sample obtained from the P(3HB) homopolymer. In the vast majority of cases, these parameters were higher for all films composed of copolymer PHA. The absolute values of the arithmetic mean (Sa) and root mean square roughness (Sq), as well as the maximum height (Sz), integrating the difference between the minimum and maximum of profile irregularities, were significantly higher for copolymer films, while differing significantly depending on the chemical composition of the PHA. The value of the arithmetic mean roughness for P(3HB-co-3HV) samples was 297 nm with the minimum inclusion of 3HV monomers (10.5 mol.%); at 32.2 mol.%, 3HV is slightly lower (206.8 nm). For 3(HB-co-4HB) copolymers, the Ra values were close (243.1–290.1 nm) and did not depend on the content of 4HB monomers. On the contrary, the presence of 3HH monomers affected the roughness of P(3HB-co-3HH) films; the value of Ra was 93.7 and 202 nm when the content of 3HHx monomers in this type of copolymer was, respectively, 10.0 and 38.0 mol.%. Thus, the chemical composition of the PHA affects the roughness of the available films.

The data obtained are consistent with the results of other studies, which indicate the effect of the composition of monomers in PHAs on the microstructure of the film surface. For example, atomic-force microscopic analysis showed that the surface roughness values of all films from P(3HB-co-3HV) with 26 and 12 mol.% HV were 92.5 and 290.8 nm, and when polyethylene glycol was added, it increased to 588.8 nm [73]. It was shown in [74] that the inclusion of 3HV monomers increases the film’s surface roughness. At the same time, there is evidence that an increase in the content of 4HB monomers in another copolymer, P(3HB-co-4HB), is accompanied by a decrease in the surface roughness of the films, which become smoother [75,76]. It should be noted that even small changes in the surface profile can lead to a wide range of changes in the cellular response, from a slight increase in cellular activity to its significant suppression. However, this effect is not universal, and different cell types differ in their sensitivity to variations in surface roughness and topography, but this requires specific studies in cultures of eukaryotic cells of various origin in vitro.

**Figure 3:**
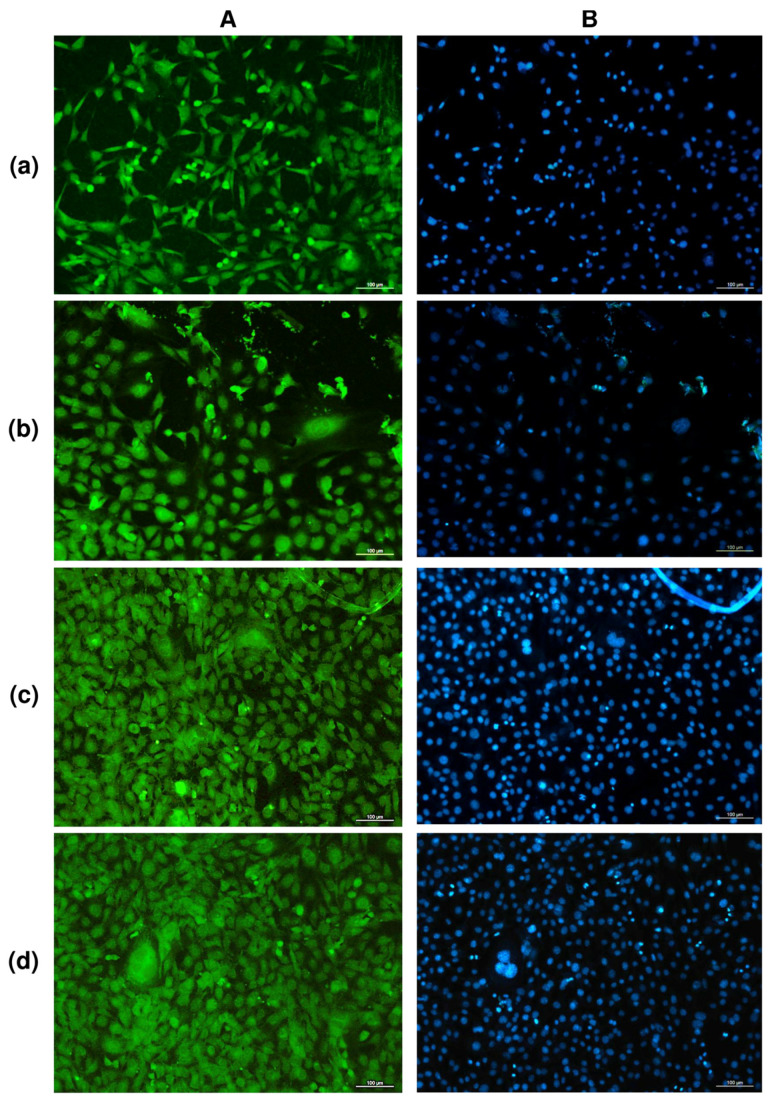
FITC (**A**) and DAPI (**B**) staining of fibroblasts NIH 3T3 on PHA films of various compositions: (**a**)—P(3HB); (**b**)—P(3HB-co-32.2 mol.% 3HV; (**c**)—P(3HB-co-35.5 mol.% 4HB; (**d**)—P(3HB-co-38.0 mol.% 3HHx; (2-day culture) bar 100 µm.

The study of solvent-cast PHA films in cell cultures in vitro showed no negative effect of any type of film in direct contact with cultured NIH 3T3 mouse fibroblasts (Figure 3).

Counting of metabolically active cells showed that the number of NIH 3T3 mouse fibroblasts on films of P(3HB) and P(3HB-co-3HV) copolymers was comparable to the control (polystyrene), amounting to approximately 9.5 ± 0.5·10^3^ cells/cm^2^ for 48 h of growth. The number of viable fibroblasts on films produced from two other, less crystalline copolymers, P(3HB-co-4HB) and P(3HB-co-3HHx), and having lower values of the contact angle—i.e., more hydrophilic and rougher—was significantly higher at 13.69 ± 1.85 and 15.18 ± 1.63·10^3^ cells/cm^2^, respectively. These results are in good agreement with the data reported by many authors who have studied the adhesion and proliferation of different cells—osteoblasts [77], fibroblasts [78], and keratinocytes [33]—and showed that matrices and films prepared from polylactide and polylactide/polyglycolide copolymers were inferior to PHA films and matrices.

### 3.2. Production and Characteristics of PHA Ultrathin Fibers by Electrostatic Spinning

Electrospinning (electrostatic spinning) is a promising technique that can be used for fabricating micro- and ultrafine fibers and fibrous scaffolds (mats) and membranes. In the electrospinning process, ultrafine fibers are formed between two oppositely charged electrodes: one is placed in a polymer solution or melt and the counter-electrode is a collecting metal screen. Electrospinning is used to produce ultra- and nano-fine fibers and porous structures based on them from solutions and melts of polymers with different structures [79]. Fiber constructions prepared from various materials by electrospinning are promising candidates to be used as scaffolds for in vitro cell cultures and as medical devices for surgical reconstruction (wound dressings, barrier membranes for guided tissue regeneration in maxillofacial surgery, etc.). The process of electrospinning has been found to have great potential in cell and tissue engineering.

Electrospinning studies using PHAs have not been conducted until quite recently. The first papers reporting the employment of this technique to produce ultrafine fibers from poly-3-hydroxybutyrate and copolymers of 3-hydroxybutyrate with 3-hydroxyvalerate were published in 2006 [80,81]. At present, the method has been tested to prepare electrospun products of PHA copolymers [54,82,83,84,85]. 

The purpose of this study was to electrospin ultrafine fibers from PHAs with different chemical structures (P(3HB) and copolymers P(3HB-co-4HB), P(3HB-co-3HV), and P(3HB-co-3HHx)) and compare their structures and physical–mechanical and biological properties. Ultrafine fibers were electrospun from PHA solutions using a Nanon 01A automatic set-up (MECC Inc., Japan). Solutions of these polymers were used to obtain nonwoven nanomembranes formed by ultrathin fibers from destructible polyhydroxyalkanoates (PHAs). With regard to this method of molding, one of the determining parameters of the process is the properties of the polymer solution, namely its dynamic viscosity. This parameter affects the electrospinning process and the quality of the resulting products.

Polymer solutions with concentrations (C, %) from 1 wt.% to 10 wt.% were prepared from PHA samples of different chemical composition using chloroform and dichloromethane, with temperature changes from 5 to 60 °C for chloroform, and from 5 to 40 °C for dichloromethane, in temperature steps of 5 °C. For each type of PHA used, an increase in the value of dynamic viscosity with an increase in the concentration of the polymer solution was revealed. The highest values of the indicator were recorded for P(3HB) solutions. An increase in the proportion of inclusion of monomers up to 10.5 mol.% caused a pronounced decrease in the studied parameter by more than five times. The lowest rates were recorded for the P(3HB-co-4HB) sample; the viscosity values for this copolymer did not exceed 500 cP for all solutions of the studied concentrations. For the P(3HB-co-3HHx) samples, the dynamic viscosity did not exceed 1500 cP at the highest concentration used. Similar measurements were carried out for different types of PHA solutions in dichloromethane. The directly proportional dependence of the dynamic viscosity of solutions on concentration was non-linear for both types of solvents. The dynamic viscosity of PHA solutions in chloroform is 3–3.5 times higher than that of solutions based on dichloromethane. PHA solutions, in descending order of dynamic viscosity, can be arranged in the following order: P(3HB)—P(3HB-co-3HV)—P(3HB-co-3HHx)—P(3HB-co-4HB).

To determine the conditions for obtaining nonwoven nanomembranes formed by ultrathin fibers, the effect of electrospinning parameters on the morphology and physicomechanical properties of the obtained samples was studied. The main investigated electrospinning parameters were the polymer concentration in solution, solution feed rate, applied voltage, and type of receiving collector (target). Chloroform solutions with polymer concentrations varying from 1 to 10 wt.% were prepared from all types of PHAs. The syringe was fixed horizontally in the set-up, and the solution feeding rate was varied from 4 to 8 mL/h, the applied voltage from 15 to 30 kV, and the working distance from 11 to 15 cm. 

The effect of the density of the polymer solution on fiber properties was studied using the homopolymer of 3-hydroxybutyric acid, in order to avoid the influence of the chemical composition of the PHA on the electrospinning process and properties of the products. The polymer concentration directly influences the quality of the electrospun fibers. Polymer solutions of density under 2 wt.% could not yield high-quality fibers, due to their low viscosity (below 100 cP). These solutions yielded a few, very thin, ultrafine fibers and a fine spray, which consisted of microdrops rather uniformly distributed on the collector. As the P(3HB) concentrations increased, the solution viscosities increased too (from 60 to 800 cP) because the entanglement of polymer molecular chains prevented the breakup of the electrically driven jet and allowed the electrostatic stresses to further elongate the jet. Stable electrospinning of ultrafine fibers in the Nanon 01A set-up was attained with P(3HB) solutions with polymer concentrations between 2 and 8 wt.% (solution viscosity 200–800 cP). The polymer concentration significantly influenced the diameter of the ultrafine fibers, which varied from 0.45 to 3.14 µm. The viscosity of the solutions with polymer concentrations above 8 wt.% was too high (approximately 1000 cP) to allow the successful formation of ultrafine fibers. Most of the fibers were cylindrical and smooth; their surface was virtually defect-free, and there were spaces between the fibers. As the polymer concentration of the solution was increased, the range of fiber diameters became wider (between 1 μm and 3 μm). As the fiber diameter increased, spaces between fibers in the fibrous mat widened from around 2 to 10 µm and the thickness of the mats increased too (from 10 to 75 µm). P(3HB) concentrations of the solution ranging between 2 and 8 wt.% yielded good-quality fibers.

Investigation of the impact of changes in applied voltage from 15 to 30 kV on fiber morphology and diameter showed the possibility of obtaining good-quality fibers in the whole investigated range. Within this voltage range, the average fiber diameter changed from 1.25 to 2.5 µm. At voltages below 10–12 kV, no fiber formation occurred, regardless of the polymer concentration used. The surface properties of nonwoven membranes comprising ultrafine fibers of different diameters, electrospun from P(3HB) solutions of different concentrations, are listed. Nanomembranes consisting of the smallest-diameter fibers (0.45–1.29 µm) had the lowest values of the water contact angle (51.20–56.57°).

The surface properties, such as surface tension (γS) and interfacial free energy (γSL), calculated using the de Gennes equations, had the lowest values at the highest cohesive force (WSL). As the fiber diameter increased above 1.8 μm, the water contact angle increased. The membranes became more porous as the fiber diameter increased. The finest, 0.45-μm diameter, fibers showed the lowest porosity levels, approximately 43.5%. The porosity levels of the membranes formed by fibers of diameters between 0.79 and 3.14 μm were similar to each other, but they were higher than those of the mats formed by the finest fibers (within the range of 62–65%). It was found that the elongation at break (elasticity index) of the membranes increased by more than two times (from 4.5 to 10.6%) with an increase in the diameter of the ultrathin fibers that formed them in the range of 0.45–3.14 μm. However, a decrease in mechanical strength was noted. Thus, the stress at break decreased from 23.16 to 6.65 MPa; at the same time, the second strength index (Young’s modulus) also dropped (from 1.16 to 0.3 GPa). These parameters of P(3HB) membranes are close to those recorded for polyethylene (0.2 GPa) and polypropylene (1.5–2.0 GPa), respectively. It should be noted that the physical and mechanical properties of the ESF of nanomembranes composed of P(3HB) generally correspond to the characteristics of human tendons in terms of Young’s modulus (0.06 GPa).

Comparison of the obtained results with published ones gives the following picture. The use of a high-density P(3HB) solution had a considerable effect on the diameter of electrospun fibers, which increased from 0.45 to 3.14 μm, and this finding was in good agreement with the data reported by other authors. An effect of the fiber diameter on the tensile strength and tensile modulus was reported for fibers prepared by electrospinning from different materials, such as caprolactone fibers [86,87]. The data reported on other polymers are contradictory: some authors reported an increase in fiber diameter with increasing voltage for polystyrene [88] and polylactide [89], and polyethylene oxide monofibers [90], while others observed the opposite results. Results showing the favorable effect of a higher applied voltage on fiber diameter are consistent with the data reported by Tong et al. [82] for fibers produced by electrospinning from P(3HB-co-3HV). Cheng et al. [91], however, reported different results: the diameter of the fibers prepared by electrospinning from P(3HB-co-3HHx) did not change as the applied voltage was increased. We also studied the effect of the polymer solution feeding rate on fiber properties. As the solution feeding rate was increased, the fiber diameter increased. A similar, though less pronounced, effect was reported by Tong et al. [82] for electrospun P(3HB-co-3HV) fibers. Variations in the working distance from 15 to 30 cm in the Nanon setup did not influence the quality and properties of electrospun P(3HB) fibers. However, they reported a significant decrease in fiber diameter (from 5 to 2 μm) with an increase in the working distance from 12 to 22 cm; this result is consistent with the observation made in [92], for electrospinning from polyacrylonitrile solutions. The results indicate the complexity of the electrospinning process and the multifactorial influence on the characteristics of the resulting products.

**Figure 4:**
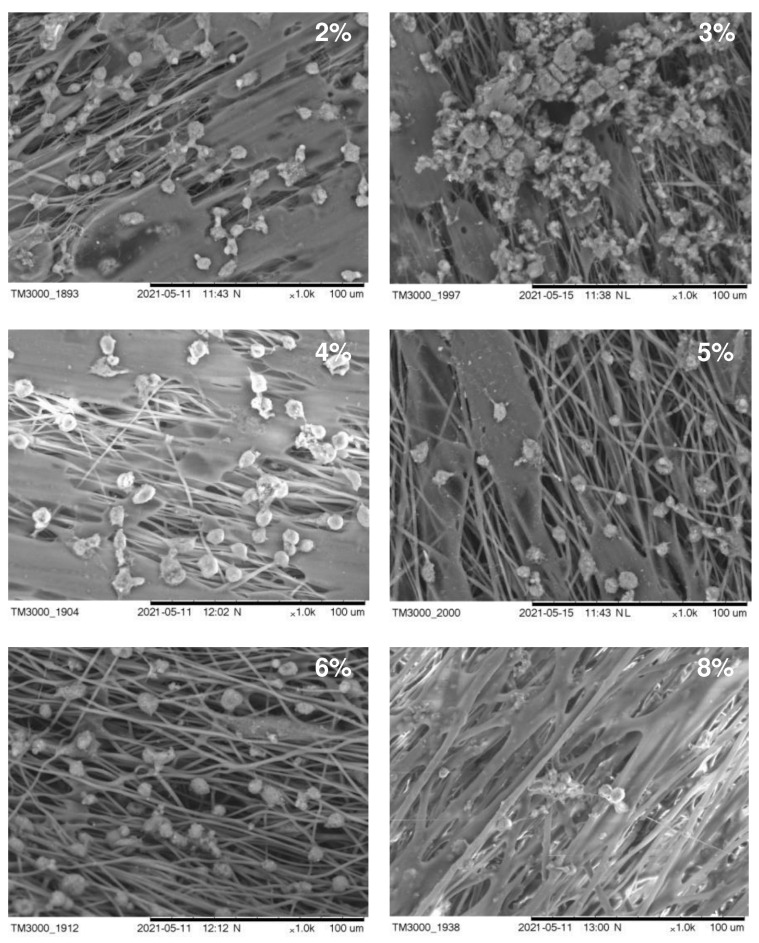
SEM images of NIH 3T3 mouse fibroblasts cultured on nanomembranes obtained by electrostatic molding from P(3HB) solutions of various concentrations.

The structure of the carriers (matrices) used in cell technologies affects the viability and functionality of the cultured cells. Therefore, the structure of the carrier must ensure the attachment, growth, and development of cells. An important characteristic in this case is also the hydrophilicity of the surface of the material, which affects cell adhesion and proliferation. In Figure 4, we present SEM images of fibroblasts from a 3-day-old culture grown on nonwoven polymer nanomembranes formed by fibers of various diameters and obtained from P(3HB) polymer solutions of various concentrations. The highest number of metabolically active cells (8–9 ± 0.85·10^3^ cells/cm^2^) was registered on nanomembranes obtained from 2–4% polymer solutions. The number of cells on nanomembrane samples obtained from denser polymer solutions and formed by larger-diameter fibers was significantly lower (approximately 6–7 ± 0.56·10^3^ cells/cm^2^).

A sign of the functional suitability of matrices is the ability of cells growing on their surface to synthesize proteins of the extracellular substance. SEM images of cells showed the formation of an extracellular substance on all samples of the studied P(3HB) nanomembranes. Particularly significant deposits were seen on samples obtained from 2–4% polymer solutions, in which the diameter of the ultrathin fibers ranged from 0.45 to 1.80 µm. This indicates the high metabolic activity of fibroblasts. On ESF nanomembranes formed by fibers with a diameter of 2.02–3.14 μm and obtained from more concentrated polymer solutions (6–8%), the secretion of extracellular deposits was recorded with an orientation along the fibers.

In general, regardless of the fiber diameter, nonwoven nanomatrices obtained from P(3HB) are favorable for the growth of fibroblast cells.

**Figure 5:**
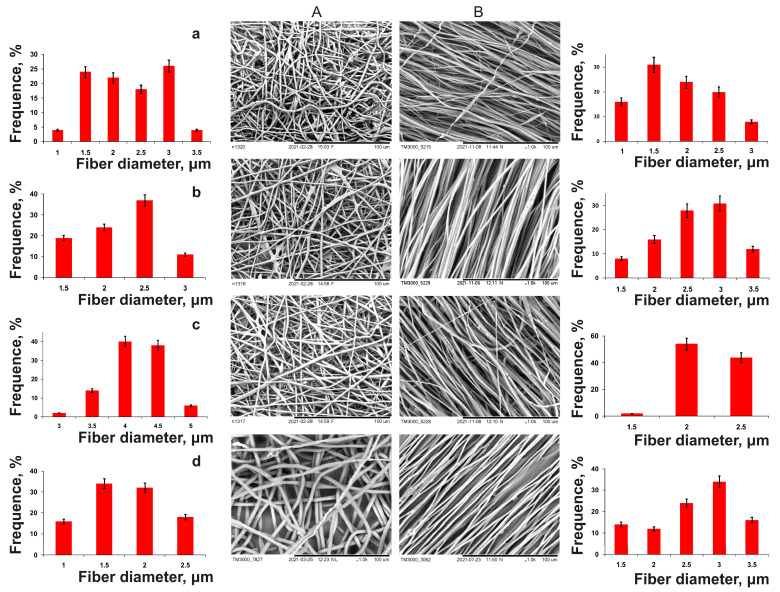
SEM images of randomly oriented (**A**) and aligned nanofibers (**B**) produced by electrospinning from solutions of PHAs with different chemical compositions: (**a**)—P(3HB); (**b**)—P(3HB-co-32.2 mol.% 3HV; (**c**)—P(3HB-co-35.5 mol.% 4HB; (**d**)—P(3HB-co-38.0 mol.% 3HHx.

Next, a family of ESF polymer nanomembranes from PHAs of various compositions was obtained, differing both in the diameter of the fibers and the structure of nonwoven matrices, and in their mechanical properties (Figure 5, Table 3). The formation of PHA solutions of various compositions in chloroform (5%) was carried out under the following conditions: needle diameter—1 mm, applied voltage—30 kV, solution supply rate—5 mL/h, working distance—15 cm, collector—flat steel plate and drum with a rotation speed of 1000 rpm, respectively. These were used for the manufacture of non-oriented and oriented ultrafine fibers.

In essence, all nanomembranes with different fiber orientations were of good quality, but differed in fiber diameter. The average fiber diameter in nonoriented membranes obtained from P(3HB) was 2.9 µm, and the membrane thickness was 30 µm; that from P(3HB-co-3HV) was 2.3 µm, and for P(3HB-co-4HB), it was significantly lower (1.7 µm). On the contrary, the diameter of the fibers also obtained from the P(3HB-co-3HHx) solution was two times higher (4.2 μm). The results of a comparison of the physical and mechanical characteristics of ES samples from PHAs of various chemical compositions (Table 3) showed that, regardless of the chemical composition, all copolymer nanomembranes had higher elongation at break, which indicated their increased elasticity, while they were characterized by a dramatic decrease in strength in terms of two indicators (Young’s modulus and breaking load). The highest values of these indices are typical for nonoriented membranes composed of P(3HB), 356.23 ± 40.62 and 9.32 ± 2.54 MPa, respectively. The presence of the 4HB monomer in the PHA reduced the mechanical strength of the membranes to the greatest extent compared to the 3HV and 3HHx monomers, according to the Young’s modulus, to 1.59 ± 0.30 and 1.22 ± 0.36 MPa; in terms of tensile strength, they were reduced by up to 0.42 ± 0.08 and 1.46 ± 0.21 MPa (with different content of 4HB monomers, respectively, 10.0 and 35.5 mol.%). The reverse effect of the composition of monomers was found when measuring the elongation at break of nanomembranes (an index of elasticity of polymer products). This indicator was the minimum, 13.3 ± 3.11%, for membranes from P(3HB), and was significantly higher for all copolymer products: 2.0–2.5 times higher for membranes from P(3HB-co-3HV) and more significantly (from 5–7 to 13 times) for membranes obtained from P(3HB-co-3HHx) and P(3HB-co-4HB), respectively. 

Oriented nanomembranes obtained from a similar set of PHAs using a rotating drum as a receiving target had significant differences from nonorientated ones in all studied characteristics (Table 3). The oriented fibers obtained from P(3HB) had an average diameter of 2.1 µm; the diameter of copolymer-oriented fibers, in contrast to nonoriented ones, had similar values. Fibers from the P(3HB-co-3HV) copolymer were comparable in diameter to nonoriented fibers (around 2.5 μm); the average diameter of fibers from P(3HB-co-3HHx) was 2.2 µm, and that of P(3HB-co-4HB) was 2.7 µm. The fiber diameter distribution width for all oriented samples was similar (2 μm). This is comparable to that for nonoriented samples. The thickness of the ESF membranes formed by oriented fibers was 25–30 µm, which is comparable to that for nonoriented fibers.

The first aspect that distinguishes oriented nanomembranes from nonoriented ones is the significantly higher mechanical strength values. This is indicative for all studied samples. The highest values of tensile strength and Young’s modulus were in homopolymer-oriented nanomembranes, respectively, 525.81 ± 1.06 and 15.17 ± 2.33 MPa, with also an increased elongation at break of up to 19.90 ± 3.2%. For copolymer-oriented nanomembranes, with an increase in the fraction of the second monomer in the copolymer PHA, an increase in the mechanical strength indices was recorded, and this distinguishes the effect of the PHA composition on the characteristics of nonoriented nanomembranes. According to the degree of influence of the second monomer in the PHA on the mechanical strength, the monomers can be arranged in the following order: 3HH—3HV—4HB. The highest values of tensile strength (431.04 ± 38.42 and 479.95 ± 52.62 MPa) were observed for the samples produced from P(3HB-co-3HHx) copolymers; they were somewhat lower (about 1.4–1.5 times) for samples from P(3HB-co-3HV) and the lowest (143.70 ± 22.30 and 195.50 ± 8.22 MPa) for samples from P(3HB-co-4HB). The next revealed difference between oriented membranes and nonoriented ones was the higher elasticity indices in all obtained samples. The highest values of elongation at break (272.46 ± 6.32 and 345.90 ± 20.54%) were obtained for P(3HB-co-4HB) samples, which were similar to nonoriented membranes.

In contrast to the results of a significant change in the physical and mechanical characteristics of nanomembranes from various types of PHA, no significant effect of the PHA composition and orientation on the hydrophilicity and surface properties of the studied polymer products was found (Table 3). The value of water contact angle, and all indicators calculated on its basis (free surface energy, free interface energy, cohesive force), for the studied representative series of polymer products had similar indicators. Analyzing the obtained results, we can conclude that, according to the degree of influence on the physical and mechanical characteristics, the studied parameters can be arranged in the following series: fiber orientation—chemical composition of PHA—concentration of polymer solution. Thus, using different types of PHA and a receiving target, it is possible to obtain ESF nanomembranes with different mechanical strength and elasticity.

PHAs are a family of polymers of different chemical composition, which differ significantly in their basic physicochemical properties, while the properties depend not only on the set but also on the ratio of monomers, and there are wide opportunities for obtaining polymer products for various applications with different functional properties. In the present work, the ESF method was simultaneously used to obtain nanomembranes from P(3HB) and three types of copolymers, in which 3HV, 4HB, and 3HHx monomers were present in macroinclusions (10 and approximately 30 mol.%).

In connection with the great prospects of the method of electrostatic formation, several types of electrospinning processes have been described, which are used to produce fibers and membranes from both synthetic and natural polymers, such as gelatin, carboxymethyl cellulose, polyethylene oxide, polylactide, polyurethanes, polyvinyl alcohol, polytrimethyl terephthalate, dimethylformamide, etc. [93,94,95,96,97,98,99]. The possibility of obtaining polymer products from various types of PHA by electrostatic molding is also discussed in the literature. The first works were carried out relatively recently (2006) using P(3HB) homopolymer and P(3HB-co-3HV) copolymer [80,81]. Further, publications appeared on the effect of process parameters on the properties of ESF products, including P(3HB-co-3HV) copolymers [82,83,100]. Later, the possibility of obtaining nanomembranes by electrospinning from PHA solutions of various compositions was studied. In [83], nanomembranes with different orientations of ultrathin fibers were obtained from P(3HB) and three types of copolymers containing monomers 3HV, 4HB, and 3HHx; the authors studied the surface microstructure, physical and mechanical properties, and attachment and development of eukaryotic cells on copolymer samples with the same content of second monomers (around 10 mol.%). The authors of [101] described the results of a comparison of three types of electrospun PHA scaffolds, P(3HB), and two types of copolymers, namely P(3HB-co-4HB) with 4HB monomer content of 7 and 97 mol.%, and P(3HB-co-3HHx) with 3HH monomer content of 5 mol.%. The authors described the morphology and diameter of the fibers, as well as some mechanical properties (Young’s modulus), depending on the type and molecular weight of PHA; the influence of the molar fraction of 3HHx monomers on the characteristics of the resulting ESF products was shown. A similar effect of 3HHx monomers in P(3HB-co-3HHx) copolymers on the properties of products formed by electrospinning has also been shown in other works [85,91,102]; samples of copolymers of this type were studied and it was shown that with an increase in 3HHx monomers from 4 to 8 mol.%, there is an increase in Young’s modulus but a decrease in elongation at break. The authors concluded that an increase in the content of 3HHx monomers does not seem to affect the mechanical properties, because the influence of the morphology of the spun fibers affects the properties of the products more significantly than the content of 3HHx monomers—that is, the chemical composition of the polymers. It should be noted that in most publications on the electrospinning of polymers of the PHA family, copolymers with very low inclusions of monomers other than 3HB (less than 10 mol.%) were studied, in contrast to the present work, in which we investigated samples of PHA copolymers, with the content of the second monomers being 10 and 30 mol.%, which certainly a broader discussion more difficult.

### 3.3. A Study of the Biological Compatibility of PHA Films and Nanomembranes in Cell Cultures In Vitro

**Figure 6:**
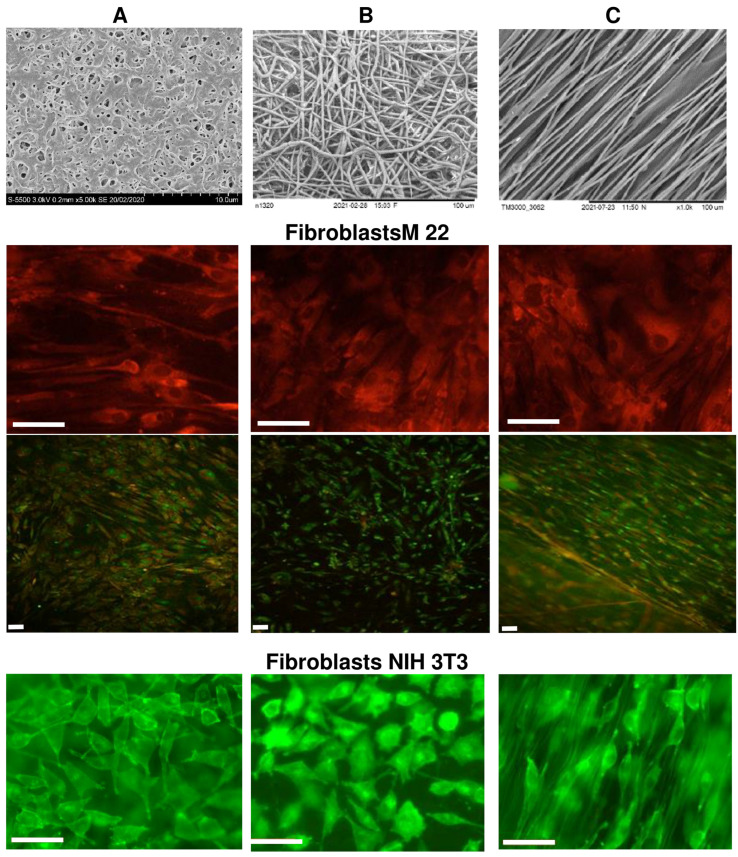
Fibroblasts cultivated on P(3HB-co-4HB) polymer carriers: (**A**)—solvent-cast film; (**B**)—random nanomembranes; (**C**)—aligned nanomembranes. Supravital staining with fluorochrome stains: fibroblasts M22 tripaflavin and rhodamine C; fibroblasts NIH 3T3-FITC; bar 50 µm.

The biological potential of polymer-cast films and nonwoven nanomatrices as cell carriers was studied using the example of three types of polymer products obtained from P(3HB-co-4HB) copolymers, in which the content of 4HB monomers was 30 mol.%. The choice is justified by the properties of this type of copolymer from the PHA family, which has the lowest degree of crystallinity and provides products with the highest elasticity [9,36,37,40]. SEM images of P(3HB-co-4HB) samples of cast films and nonwoven membranes formed by oriented and nonoriented ultrathin fibers, studied in eukaryotic cell cultures, are shown in Figure 6.

Nonwoven membranes obtained by the method of electrostatic molding of polymer solutions, as noted above, had differences in structure and physical and mechanical properties. The nonwoven membranes had contact angles (on the order of 51–68°) that were much lower than those of the solvent-cast films (up to 92°). This was accompanied by differences in the values of surface tension and the energy characteristics of the surface, as well as the physical and mechanical properties of polymer products obtained by the two methods studied. This was especially evident when measuring the elongation at break (a measure of the elasticity of products). Oriented membranes had higher values of mechanical strength, characterized by the values of Young’s modulus and tensile strength, compared to nonoriented membranes and films, as well as the highest values of elongation at break (Table 3).

Upon direct contact with the cells of the studied samples of P(3HB-co-4HB) matrices of all types, there were no signs of cytotoxicity in cultures of M22 human fibroblasts and NIH 3T3 mouse fibroblasts (Figure 5). Human M22 fibroblasts were transparent, clearly defined, and had a morphology characteristic of the culture—they did not contain inclusions and retained their original structural integrity. The proliferative activity of the cells did not undergo any visible changes during the entire period of cultivation. The process of colonization of all coating samples with M22 cells was very pronounced, starting from the third day of cultivation. Thus, on smooth films (sample 1), individual cells were found already after 1 day of culturing products; after 2 days, their number was 2.5 ± 0.14·10^3^ cells/cm^2^; after 3 days, it was 5 ± 0.23·10^3^ cells/cm^2^; after 5 days, it was 15 ± 0.86·10^3^ cells/cm^2^; after 7 days, it was 20 ± 0.74·10^3^ cells/cm^2^. After a week of cultivation, a complete confluent (occupying more than 80% of the entire surface) monolayer of cells was formed on the surface of the samples. The cells had a characteristic flattened or fibroblast-like—and, less often, polygonal—shape, and were biologically complete. The dynamics of the cell populations of oriented and unoriented nanomembranes (samples 2 and 3) were lower. Individual cells were found on the membrane surface on the third day of cultivation; after 5 days, their amount was 8 ± 0.45·10^3^ cells/cm^2^; after 7 days, it was 16 ± 0.74·10^3^ cells/cm^2^ (subconfluent monolayer); most of the cells were not flattened but spindle-shaped, while maintaining the integrity of their internal composition. The maximum population of films and membranes was observed on the 7–8th day; then, the number of cells began to gradually decrease until the 10th day, when the number of cells on the films decreased by 25%; on the membranes, it decreased more significantly, by 30–40%. Starting from the 11th day, there was a slight increase in the number of cells in all samples. By the end of the 12th day, the number of cells reached the level of 18 ± 0.61·10^3^ cells/cm^2^ on films and 12 ± 0.38·10^3^ cells/cm^2^ on membranes. At the same time, the vast majority of attached cells had a characteristic fibroblast-like shape. Thus, with standard culture methods, samples provide the long-term preservation of attached cells.

The biological usefulness of the attached cells, determined by the cell membrane integrity (ICM) parameter, was maintained for 7–8 days from the start of colonization at a high level: 36.0 points for smooth films and 36.5 points for oriented and nonoriented nonwoven membranes (at a normal rate of 28–42 points). A slight decrease in the ICM value of attached cells (30.0 points) was noted on the 10–11th day of cultivation. At this stage of the study, the maximum content of damaged cells (20%) was noted in the cell population. By 12–14 days, the ICM values increased slightly and amounted to 34.6 points. At the same time, the proportion of cells with impaired membrane components (10%) was higher than on days 7–8 (5%). This confirms the assumption that an adequate period of cell cultivation on the studied samples as skin biografts should not exceed 7–8 days. It is important to note that the biological usefulness of the cells was maintained for 7–8 days after the colonization of all samples.

The results of the proliferative activity of fibroblasts, including the proliferation index (PI), are presented in Table 4, from which it follows that these indicators on the three studied samples of experimental cell matrices were comparable.

The morphology of NIH 3T3 mouse fibroblasts cultured on all samples had minor differences, but no visible negative changes. The results of the quantitative determination of cells stained with fluorescent dyes showed the influence of the orientation of ultrafine fibers on the location and number of cells. Fibroblasts grown on oriented fiber membranes were distributed along the fibers and were predominantly spindle-shaped. On unoriented membranes, cells were also distributed along the fibers, while triangular cells predominated, with 3–4 processes, with the help of which the cells were attached to the fibers. The number of cells on unoriented nanomembranes was the highest and amounted to around 6.41 ± 0.20·10^3^ cells/cm^2^. The number of viable fibroblasts on films and oriented nanomembranes was lower than on nonoriented ones, 4.61 ± 0.18·10^3^ and 3.85 ± 0.28·10^3^ cells/cm^2^, respectively, but generally comparable to or slightly higher than the results in the control (culture plates, polystyrene). This agrees with the results previously obtained in the culture of mouse fibroblasts of this line, which were grown on nanomembranes with different fiber orientations, but obtained from the P(3HB-co-4HB) copolymer with a significantly lower content of 4HB monomers (approximately 10 mol.%) [83]. It was shown in the work that the number of cells on nonoriented membranes was the highest, but, in general, for the same period of cultivation, the number of cells was lower (1.6–1.8 and 2.2–2.4·10^3^ cells/cm^2^), respectively, on oriented and nonoriented nanomembranes.

Thus, all types of PHA polymer scaffolds studied are biocompatible and suitable for cell cultivation; however, nonoriented nonwoven membranes are most favorable for the studied NIH 3T3 mouse fibroblast line. The revealed effect of the orientation of ultrathin fibers in PHA nanomatrices on the attachment and development of fibroblasts is consistent with the data reported by other authors [103,104] suggesting that the fiber orientation influences cell morphology irrespective of PHA composition. Which orientation—aligned or random—is more favorable depends on the type of cells. Aligned fibers are more advantageous for the growth and development of osteoblasts, vascular endothelial cells, and neurons than randomly oriented ones [82,85,105]. Fibroblast cells, however, are rather affected by the porosity of the scaffold than by its being aligned or randomly oriented [103,106].

### 3.4. An In Vivo Study of P(3HB-co-4HB) Films and Nanomembranes as Experimental Wound Dressings

An important and socially significant task of reconstructive medicine is focused on obtaining new materials for the development of effective agents to repair damage to the skin. This is due to the constant increase in the number of skin defects due to burns, injuries, and surgical interventions. The range of surgical and therapeutic agents used to close and restore skin defects, as well as materials and medicines used for their manufacture, is large and includes hundreds of items [107,108]. The principles and methods of treatment of skin wounds depend on many factors: the depth and severity of the injury and the phase of the wound process, the localization of the wound and the presence of infection, the patient’s comorbidities, and the medications taken by the patient. The main principle of treatment is wound cleansing and the creation of optimal conditions for regeneration.

The actual direction of research is the design of atraumatic wound coverings using new materials. Non-stick dressings are based on the principle of using a hydrophobic polymeric material or the formation of a hydrophobic layer on the dressing adjacent to the wound [109]. Absorbable coatings meet biomedical requirements to the greatest extent and can be useful both in the early stages of wound and burn treatment and in later stages. The development of absorbable polymer coatings with different biodegradation periods is currently an important direction in the field of creating effective coatings for wounds and burns. This direction has not yet gained popularity due to the limited availability of biodegradable materials that meet the necessary requirements for materials used for wound dressings. To improve wound dressings, an active search for new functional materials and methods for their processing is being carried out.

The analysis of the literature and the results obtained indicate the high potential of biodegradable polyesters of hydroxyalkanoic acids, which, unlike polylactide, do not swell and do not hydrolyze in an aqueous medium, so they do not sharply acidify tissues during degradation. They are thermostable; therefore, they are sterilized by generally accepted methods; they are permeable to water vapor and oxygen, they are biocompatible, and they do not cause local irritation or allergic effects. To date, PHAs and their composites are thought to have good potential as emerging materials for medical devices such as sutures, bone plates, surgical mesh, and cardiovascular patches, to name a few [37]. A major breakthrough for PHAs as a new class of biomaterials is the clearance obtained from the Food and Drug Administration of the United States of America for the use of P(4HB)-derived TephaFLEX absorbable sutures [110]. These biopolymers are also promising for processing into a hydrophobic coating that does not stick to the wound, in the form of flexible films and/or membranes formed by ultrathin polymer fibers; due to their transparency, they make it possible to observe the wound and thus to introduce drugs into PHA products and load them with proliferating cells [54].

Experimental polymer products in the form of cast films and nanomembranes constructed from P(3HB-co-4HB) copolymer and coated with type 1 collagen were studied as resorbable wound dressings on aseptic model defects of the skin of laboratory animals.

In the postoperative period, all animals were healthy and active, with normal feeding behavior, and moved independently. After waking the animals from anesthesia, no signs of pain were recorded. The results of the evaluation of the dynamics of skin wound healing during the experiment are presented in Table 5. Measurements of the area of wounds in the dynamics of observation showed that, in all groups of animals, the defects healed, while, in the case of wound closure with both types of nanomembranes, healing occurred almost equally and exceeded the result observed under the films.

On the 7th day in the control group, all animals retained an epidermal defect measuring around 0.5 cm^2^ (0.55% of the initial wound), with inflammatory and infiltrative signs. In the incompletely restored dermis, insignificant foci of inflammation were visible, and subcutaneous adipose tissue with gland ducts was pronounced. Measurement of the surface area of the wound defect in the experimental groups showed that, during these periods, the decrease in the area of the wound surface was the most significant in the group with the use of nonoriented nanomembranes, under which the area of the wounds decreased by 7.2 times. In the group of animals receiving oriented nanomembranes, the area of wounds decreased somewhat less, by 4.2 times; in the group treated with bulk films, it was even smaller (by 2.7 times) (Table 4).

**Figure 7:**
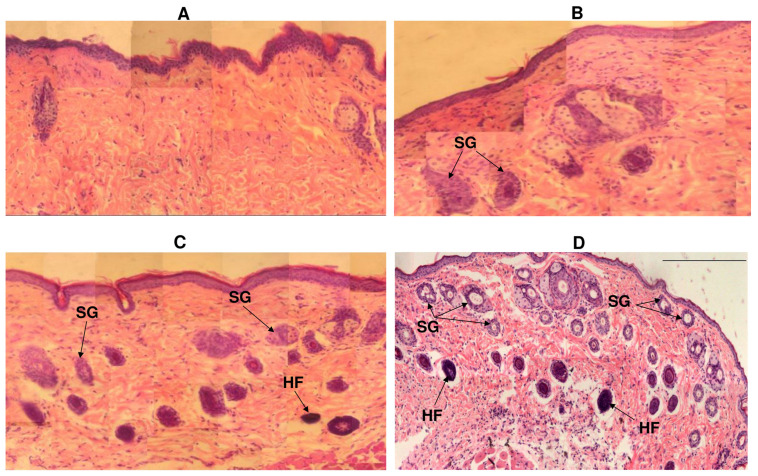
Histological sections from the skin defect site under different types of wound dressings: (**A**)—control; (**B**)—solvent-cast film; (**C**)—aligned nanomembrane; (**D**)—random nanomembrane. Notations: SG—sebaceous glands, HF—hair follicles. Hematoxylin and eosin staining, 100×. Wound healing (day 10 after defect creation).

By the 10th day, almost complete wound re-epithelialization was observed in experimental animals under polymer nanomembranes of both types (Figure 7).

Experimental animals had many small capillaries in the dermis; collagen fibers were arranged in dense strands parallel to the epidermis, i.e., fibrous scars formed. In the newly formed tissue of animals, in which the defect was closed with nanomatrices, restoration of the ducts of the sebaceous glands was noted; in the dermis, the presence of hair follicles was observed. This was most pronounced in animals whose wounds were closed with nonoriented P(3HB-co-4HB) nanomembranes. By the 12th day, complete epithelialization of skin wounds was noted in all animals.

In general, the results of planimetric measurements and analysis of histological sections showed the active regeneration of skin defects in groups of animals using P(3HB-co-4HB) nanomembranes with both types of fiber orientation. This was also confirmed by counting of hair follicles, sebaceous glands, and horny cysts as objective indicators of the dynamics of skin defect recovery. Sebaceous glands and hair follicles are a source of undifferentiated epithelial cells (similar in structure to the cells of the basal layer) involved in regeneration. Horny cysts are an indicator of a slowdown in the differentiation of epidermal cells in relation to an acceleration in the proliferation of basal cells. Earlier regenerative activity of the skin was noted with the use of polymer nanomembranes.

On the 7th day in these experimental groups, the appearance of hair follicles and sebaceous glands was noted, while, in animals of the control group, only the presence of horny cysts was seen. The number of hair bulbs and sebaceous glands when closing defects with oriented and nonoriented nanomembranes on day 10 was generally close—respectively, 20–22 and 14–17—and these indicators were exceeded when closing the defect with polymer films (eight hair bulbs, four sebaceous glands); horn cysts were also present (Table 4).

An important indicator of the reparative process of the skin is the phenomenon of acanthosis—a thickening and an increase in the number of rows of spiny and granular layers with an elongation of the epidermal processes that penetrate deep into the skin itself. This process is based on the increased proliferation of basal and spiny cells (proliferative acanthosis) and slowing down of the differentiation of epidermal cells. The results of this indicator are presented in Table 4. By the 7th day of the experiment, the most pronounced proliferative acanthosis was noted in the experimental group under nonoriented nanomembranes (18 acanthotic bands with an immersion depth of 659.7 µm); under oriented nanomembranes, the performance was similar (15 acanthotic bands at a depth of 540.4 µm); under the films, the indicators lagged behind (six acanthotic bands at a depth of 389.5 µm). At this time, in the control group of animals, in which the wounds were closed with a gauze bandage, no signs of acanthosis were found. By the 10th day, regeneration of the defect was noted, with the disappearance of acanthosis and restoration of the morphological structure of the epidermis in animals in experimental groups using both types of nanomembranes. Under polymer films, defect regeneration was noted on the 12th day.

The results of planimetric measurements and analysis of histological sections showed the regeneration of model skin defects in groups of animals with the use of experimental wound dressings from P(3HB-co-4HB) of all types, but most actively when using nanomembranes obtained by electrospinning. This is consistent with the literature data. The strong functional properties of P(3HB-co-4HB) copolymers as a material for reconstructive medicine have been reported since the first examples of the use of this type of PHA in experiments on laboratory animals [111]. The authors of this work registered the high biocompatibility of this copolymer and a minimal inflammatory response of tissues to it. In [101], high biocompatibility, dynamics of biodegradation adequate for the restoration of damaged tissues, and a minimal tissue response were described when tissue engineering scaffolds obtained by electrospinning from PHA copolymers were implanted into laboratory animals (P(3HB-co-3HHx) with a 3HHx monomer content of 5 mol.% and copolymers P(3HB-co-4HB) with a 4HB monomer content of 7 and 97 mol.%. The authors found that the tissue response improved when using matrices with a higher content of 4HB monomers in the copolymer. Electroformed P(3HB-co-4HB) copolymers were evaluated as promising biomaterials due to their biodegradability, elasticity, and high biocompatibility. It was concluded that the studied hydrophobic nonwoven copolymer membranes were comparable in their mechanical characteristics to those of the skin, and therefore can be recommended for the development of tissue-engineered equivalents of wound dressings. It was also shown in [101] that the vapor permeability of the studied ESF nanomembranes significantly exceeds that of synthetic film materials (polyethylene, polypropylene, polyethylene terephthalate, etc.). The effectiveness of the P(3HB-co-4HB) copolymer in the composition of wound dressings of various types was shown in a series of studies performed on laboratory animals. For example, in [112], electrospun membranes composed of P(3HB-co-4HB) copolymer and similar nanomembranes carrying fibroblasts obtained from adipose tissue were studied on model skin defects in Wistar rats. Both types of nanomembranes promoted wound healing, vascularization, and the regeneration of newly formed tissues at the site of the defect. The use of nanomembranes with cells ensured the healing of skin wounds 1.4 times faster than wounds under cell-free membranes, and 3.5 times faster than wound healing under eschar in the control cases (without P(3HB-co-4HB) wound dressing). Hybrid wound dressings using two biomaterials (bacterial nanocellulose (BC) and P(3HB-co-4HB) as copolymers loaded with actovegin or carrying fibroblasts were studied in laboratory animals with model skin burns [113]. Evaluation of the dynamics of skin defect healing using wound planimetry, histological techniques, and molecular detection of angiogenesis factors, such as inflammation, type I collagen, and keratin 10 and 14, showed more effective wound healing under all types of experimental wound dressings based on BC/P(3HB-co-4HB) compared with the control, which used a commercial wound dressing, namely VoskoPran, based on beeswax and medicinal ointments (Biotekfarm, Russia). In [114], a positive assessment was also presented for P(3HB-co-4HB) and polycaprolactone copolymers, from a mixture of which biomimetic scaffolds were obtained by microfluidic 3D printing, carrying bone marrow stem cells and endothelial cells. These tissue-engineered scaffolds had excellent mechanical properties and a hierarchical porous structure, providing accelerated wound healing and promoting re-epithelialization, collagen deposition, and capillary formation in model wound defects in laboratory rats.

## 4. Conclusions

A set of biodegradable polyhydroxyalkanoates (PHAs) of various chemical compositions—poly-3-hydroxybutyrate homopolymer (P(3HB) and copolymers containing, in addition to 3HB monomers, macroinclusions (10 and 30 mol.%) of 3-hydroxyvalerate (3HV) and 3-hydroxyhexanoate monomers (3HHx)—were used to obtain polymer nano-products. Solvent casting films and nonwoven nanomembranes were obtained from solutions of all types of polymers by the method of solvent evaporation and the method of electrostatic formation of ultrathin fibers with different orientations. Surface structures and mechanical and biological properties of polymer products were studied depending on the method of obtaining and the composition of PHAs. In cultures of eukaryotic cells, it has been shown that all types of films and nanomembranes from PHAs are biocompatible and are cell carriers of good quality. Polymer films and nanomembranes with the highest elasticity and obtained from the least crystalline P(3HB-co-4HB) copolymers were studied in cultures of NIH 3T3 mouse fibroblasts and diploid human embryonic fibroblast cells of the M22 line and were positively evaluated as cell matrices. P(3HB-co-4HB) polymeric films, as oriented and nonoriented nanomembranes coated with type 1 collagen, were studied as wound dressings in laboratory animals with model surgical skin lesions. The results showed the regeneration of model skin defects in groups of animals using experimental P(3HB-co-4HB) wound dressings of all types, but most actively when using nonoriented nanomembranes obtained by electrospinning. This confirms the positive properties of nonwoven nanomembranes obtained by electrospinning from degradable low-crystalline copolymers P(3HB-co-4HB) as wound dressings for the healing of skin defects. The positive results obtained with the investigated types of polymeric films and nanomembranes confirm the strong potential of these nanoproducts from degradable microbial PHAs and allow the planning of more extensive in vivo studies.

## Figures and Tables

**Table 1:** Compositions and physicochemical properties of different PHAs.

Sample	PHA Composition (mol.%)		Average Molecular Weight M_w_ (kDa)	Polydispersity Ð	Degree of Crystallinity C_x_ (%)	Glass Transition Temperature T_g_ (°C)	Crystallization Temperature T_c_ (°C)	Melting Temperature T_melt_ (°C)	Thermal Degradation Temperature T_degr_ (°C)
P(3HB)—3-hydroxybutyrate homopolymer									
1	100.0		860	2.5	78	-	85	176.3	280.2
Copolymers:									
P(3HB-co-3HV)									
2	89.5	10.5	467	1.8	60	−1.0	64.2	175.1	283.9
3	72.8	32.2	576	3.2	58	−1.9	78.1	162.5	275.9
P(3HB-co-4HB)									
4	90.0	10.0	262	3.1	50	−11.7	68.2	161.4	268.0
5	64.5	35.5	660	3.6	26	−9.5	58.5	165.5	278.4
P(3HB-co-3HHx)									
6	90.0	10.0	470	2.9	60	−0.2	63.2	170.2	262.7
7	62.0	38.0	486	3.7	52	−1.6	71.2	169.2	260.1

**Table 2:** Surface properties of solvent-cast films prepared from PHAs with different compositions.

Sample	PHA Composition (mol.%)		Water Contact Angle, °	Surface Energy, mN/m	Interfacial Free Energy, γSL, erg/cm^2^	Cohesive Force, WSL, erg/cm^2^	Arithmetic Mean Surface Roughness, (Sa) nm	Root Mean Square Roughness, (Sq) nm	Peak-to-Valley Height, (Sz) nm
P(3HB)—homopolymer poly-3-hydroxybutyrate									
1	100.0		92.1 ± 6.33	30.8 ± 0.53	8.92	94.74	154.0	180.1	1255.9
Copolymers:									
P(3HB-co-3HV)									
2	89.5	10.5	64.6 ± 4.1	49.6 ± 1.27	2.23	120.23	297.0	367.4	2355.4
3	67.8	32.2	69.4 ± 9.4	50.8 ± 2.64	1.98	121.70	206.8	254.1	1594.7
P(3HB-co-4HB)									
4	90.0	10.0	73.2 ± 4.62	41.5 ± 0.89	4.38	109.98	243.1	302.5	1900.8
5	64.5	35.5	62.5 ± 5.33	44.9 ± 0.52	3.37	114.39	290.1	370.4	2321.3
P(3HB-co-3HHx)									
6	90.0	10.0	71.5 ± 1.91	48.8 ± 2.44	2.40	119.28	93.7	118.2	880.6
7	62.0	38.0	56.3 ± 6.16	57.1 ± 2.89	0.96	129.02	172.4	222.4	1677.8

**Table 3:** Surface properties and physical and mechanical characteristics of nanomembranes obtained by electrospinning and PHA solutions of various compositions.

PHA Composition, mol.%	Surface Properties				Physical and Mechanical Properties		
	Water Contact Angle (θ, °)	Free Surface Energy (γS) (erg/cm^2^)	Free Interface Energy (γSL) (erg/cm^2^)	Cohesive Force (WSL) (erg/cm^2^)	Tensile Strength (MPa)	Young’s Modulus (MPa)	Elongation at Break (%)
Nanomembranes composed of random ultrafine fibers							
P(3HB) (100mol.%)	64.33 ± 1.71	37.38	5.85	104.34	356.23 ± 40.62	9.32 ± 2.54	13.3 ± 3.11
P(3HB-co-10.5 mol.% 3HV)	67.17 ± 1.12	35.20	6.76	101.25	5.81 ± 1.06	0.67 ± 0.06	40.72 ± 6.20
P(3HB-co-32.2 mol.% 3HV)	72.63 ± 0.81	30.71	8.94	94.57	13.91 ± 2.07	0.80 ± 0.09	38.33 ± 5.34
P(3HB-co-10.0 mol.% 4HB)	72.12 ± 0.55	31.19	8.69	95.30	1.59 ± 0.30	0.42 ± 0.08	172.98 ± 10.33
P(3HB-co-35.5 mol.% 4HB)	73.23 ± 0.42	30.24	9.20	93.84	1.22 ± 0.36	1.46 ± 0.21	177.74 ± 20.67
P(3HB-co-10.0 mol.% 3HHx)	74.72 ± 1.35	29.07	9.86	92.01	18.68 ± 2.85	0.76 ± 0.10	69.45 ± 4.61
P(3HB-co-38.0 mol.% 3HHx)	69.85 ± 0.87	33.58	7.49	98.89	26.12 ± 3.48	0.78 ± 0.18	89.59 ± 7.13
Nanomembranes composed of aligned ultrafine fibers							
P(3HB) (100)	69.16 ± 1.10	34.26	6.79	103.50	525.81 ± 1.06	15.17 ± 2.33	19.90 ± 3.21
P(3HB-co-10.5 mol.% 3HV)	76.72 ± 1.35	33.07	10.36	102.01	263.52 ± 49.08	12.26 ± 2.65	51.56 ± 2.10
P(3HB-co-32.2 mol.% 3HV)	69.72 ± 1.35	31.17	12.40	101.41	321.90 ± 27.33	14.13 ± 2.05	91.16 ± 7.32
P(3HB-co-10.0 mol.% 4HB)	72.12 ± 1.05	32.04	14.06	98.01	143.70 ± 22.30	12.70 ± 0.82	272.46 ± 6.32
P(3HB-co-35.5 mol.% 4HB)	70.22 ± 1.50	30.14	12.26	100.12	195.50 ± 8.22	16.91 ± 1.56	345.90 ± 20.54
P(3HB-co-10.0 mol.% 3HHx)	66.26 ± 1.06	31.26	14.29	100.53	431.04 ± 38.42	13.26 ± 2.23	206.69 ± 3.61
P(3HB-co-38.0 mol.% 3HHx	68.16 ± 1.00	30.26	11.29	99.58	479.95 ± 52.62	15.71 ± 2.32	190.00 ± 8.66

**Table 4:** Proliferative activity of fibroblasts of the M22 line in the control (polystyrene) and cultivated on cell carriers studied experimental samples from P(3HB-co-4HB).

No. Culture Passage M22	Cell Proliferation Index for 3 Days			
	Control (Polystyrene)	Sample No.1 (Cast Film)	Sample No.2 (Nonwoven Oriented Membrane)	Sample No.3 (Nonwoven Unoriented Membrane)
1	1.83 + 0.07	1.87 + 0.09	2.08 + 0.11	1.80 + 0.11
2	2.13 + 0.11	2.14 + 0.14	2.10 + 0.8	2.08 + 0.12
3	1.96 + 0.07	1.96 + 0.09	1.96 + 0.10	1.88 + 0.10

**Table 5:** Dynamics of healing of skin defects when using wound dressings made of copolymer P(3HB-co-4HB).

Animal Groups, Type of Wound Dressing	Control (Aseptic Gauze Bandage)	Experimental Wound Dressings		
		Solvent-Cast Film	Aligned Nanomembrane	Random Nanomembrane
Wound area, cm^2^				
1 day	0.92 ± 0.019	0.9 ± 0.011	0.90 ± 0.011	0.93 ± 0.054
7 days	0.506 ± 0.027	0.34 ± 0.012	0.24 ± 0.012	0.13 ± 0.04
Average healing rate, cm^2^/day				
1–4 days	0.065 ± 0.0002	0.069 ± 0.0029	0.084 ± 0.0034	0.105 ± 0.023
4–7 days	0.085 ± 0.0013	0.159 ± 0.0024	0.215 ± 0.065	0.253 ± 0.019
Indicators of skin defect repair: number of hair bulbs, sebaceous glands, and horn cysts				
7 days	-	8 horn cysts	5 hair bulbs 7 sebaceous glands	8 hair bulbs 10 sebaceous glands
10 days	6 hair bulbs 4 sebaceous glands 8 horn cysts	8 hair bulbs 4 sebaceous glands 4 horn cysts	20 hair bulbs 14 sebaceous glands 0 horn cysts	22 hair bulbs 17 sebaceous glands 0 horn cysts
Number of acanthotic bands and their average depth of immersion in the dermis				
7 days	7 acanthotic bands 295.6 µm	6 acanthotic bands 389.5 µm	15 acanthotic bands 540.4 µm	18 acanthotic bands 659.7 µm
10 days	16 acanthotic bands 324.7 µm	no acanthosis	no acanthosis	no acanthosis

«-»—indicators of skin defect repair are missing.

## Data Availability

Not applicable.

## References

[1] García M.C., Quiroz F. (2018). Nanostructured Polymers. Nanobiomaterials.

[2] Tang Z., He C., Tian H., Ding J., Hsiao B.S., Chu B., Chen X. (2016). Polymeric Nanostructured Materials for Biomedical Applications. Prog. Polym. Sci..

[3] Adabi M., Naghibzadeh M., Adabi M., Zarrinfard M.A., Esnaashari S.S., Seifalian A.M., Faridi-Majidi R., Tanimowo Aiyelabegan H., Ghanbari H. (2017). Biocompatibility and Nanostructured Materials: Applications in Nanomedicine. Artif. Cells Nanomed. Biotechnol..

[4] Fratoddi I., Bearzotti A., Venditti I., Cametti C., Russo M.V. (2016). Role of Nanostructured Polymers on the Improvement of Electrical Response-Based Relative Humidity Sensors. Sens. Actuators B Chem..

[5] Stevanović M. (2019). Biomedical Applications of Nanostructured Polymeric Materials. Nanostructured Polymer Composites for Biomedical Applications.

[6] Koller M. (2018). Biodegradable and Biocompatible Polyhydroxy-Alkanoates (PHA): Auspicious Microbial Macromolecules for Pharmaceutical and Therapeutic Applications. Molecules.

[7] Kulikouskaya V.I., Nikalaichuk V.V., Bonartsev A.P., Chyshankou I.G., Akoulina E.A., Demianova I.V., Bonartseva G.A., Hileuskaya K.S., Voinova V.v. (2022). Fabrication of Microstructured Poly(3-Hydroxybutyrate) Films with Controlled Surface Topography. Proc. Natl. Acad. Sci. Belarus Chem. Ser..

[8] Afshar A., Gultekinoglu M., Edirisinghe M. (2022). Binary Polymer Systems for Biomedical Applications. Int. Mater. Rev..

[9] Puppi D., Pecorini G., Chiellini F. (2019). Biomedical Processing of Polyhydroxyalkanoates. Bioengineering.

[10] Shi J., Votruba A.R., Farokhzad O.C., Langer R. (2010). Nanotechnology in Drug Delivery and Tissue Engineering: From Discovery to Applications. Nano Lett..

[11] Hench L., Jones J. (2005). Biomaterials, Artificial Organs and Tissue Engineering.

[12] Nair L.S., Laurencin C.T. (2007). Biodegradable Polymers as Biomaterials. Prog. Polym. Sci..

[13] Viera Rey D.F., St-Pierre J.-P. (2019). Fabrication Techniques of Tissue Engineering Scaffolds. Handbook of Tissue Engineering Scaffolds: Volume One.

[14] Eltom A., Zhong G., Muhammad A. (2019). Scaffold Techniques and Designs in Tissue Engineering Functions and Purposes: A Review. Adv. Mater. Sci. Eng..

[15] Nazarnezhad S., Baino F., Kim H.-W., Webster T.J., Kargozar S. (2020). Electrospun Nanofibers for Improved Angiogenesis: Promises for Tissue Engineering Applications. Nanomaterials.

[16] Volkov A.V., Muraev A.A., Zharkova I.I., Voinova V.V., Akoulina E.A., Zhuikov V.A., Khaydapova D.D., Chesnokova D.V., Menshikh K.A., Dudun A.A. (2020). Poly(3-Hydroxybutyrate)/Hydroxyapatite/Alginate Scaffolds Seeded with Mesenchymal Stem Cells Enhance the Regeneration of Critical-Sized Bone Defect. Mater. Sci. Eng. C.

[17] Sampath U., Ching Y., Chuah C., Sabariah J., Lin P.-C. (2016). Fabrication of Porous Materials from Natural/Synthetic Biopolymers and Their Composites. Materials.

[18] Fitzsimmons R.E.B., Mazurek M.S., Soos A., Simmons C.A. (2018). Mesenchymal Stromal/Stem Cells in Regenerative Medicine and Tissue Engineering. Stem Cells Int..

[19] Wang M., Chen L.J., Ni J., Weng J., Yue C.Y. (2001). Manufacture and Evaluation of Bioactive and Biodegradable Materials and Scaffolds for Tissue Engineering. J. Mater. Sci. Mater. Med..

[20] Velema J., Kaplan D. (2006). Biopolymer-Based Biomaterials as Scaffolds for Tissue Engineering. Tissue Engineering I.

[21] Hosseinkhani H., Hosseinkhani M. (2009). Tissue Engineered Scaffolds for Stem Cells and Regenerative Medicine. Trends in Stem Cell Biology and Technology.

[22] Park J.B., Lakes R.S. (2007). Tissue Response to Implants. Biomaterials.

[23] Kulikouskaya V.I., Nikalaichuk V.V., Bonartsev A.P., Akoulina E.A., Belishev N.V., Demianova I.V., Chesnokova D.V., Makhina T.K., Bonartseva G.A., Shaitan K.V. (2022). Honeycomb-Structured Porous Films from Poly(3-Hydroxybutyrate) and Poly(3-Hydroxybutyrate-Co-3-Hydroxyvalerate): Physicochemical Characterization and Mesenchymal Stem Cells Behavior. Polymers.

[24] Meyer U., Büchter A., Wiesmann H., Joos U., Jones D. (2005). Basic Reactions of Osteoblasts on Structured Material Surfaces. Eur. Cell. Mater..

[25] Wei J., Yoshinari M., Takemoto S., Hattori M., Kawada E., Liu B., Oda Y. (2007). Adhesion of Mouse Fibroblasts on Hexamethyldisiloxane Surfaces with Wide Range of Wettability. J. Biomed. Mater. Res. B Appl. Biomater..

[26] Jansen E.J.P., Sladek R.E.J., Bahar H., Yaffe A., Gijbels M.J., Kuijer R., Bulstra S.K., Guldemond N.A., Binderman I., Koole L.H. (2005). Hydrophobicity as a Design Criterion for Polymer Scaffolds in Bone Tissue Engineering. Biomaterials.

[27] Tezcaner A., Bugra K., Hasırcı V. (2003). Retinal Pigment Epithelium Cell Culture on Surface Modified Poly(Hydroxybutyrate-Co-Hydroxyvalerate) Thin Films. Biomaterials.

[28] Pompe T., Keller K., Mothes G., Nitschke M., Teese M., Zimmermann R., Werner C. (2007). Surface Modification of Poly(Hydroxybutyrate) Films to Control Cell–Matrix Adhesion. Biomaterials.

[29] Lucchesi C., Ferreira B.M.P., Duek E.A.R., Santos A.R., Joazeiro P.P. (2008). Increased Response of Vero Cells to PHBV Matrices Treated by Plasma. J. Mater. Sci. Mater. Med..

[30] Grøndahl L., Chandler-Temple A., Trau M. (2005). Polymeric Grafting of Acrylic Acid onto Poly(3-Hydroxybutyrate- *c o* -3-Hydroxyvalerate): Surface Functionalization for Tissue Engineering Applications. Biomacromolecules.

[31] Hu S.-G., Jou C.-H., Yang M.C. (2003). Protein Adsorption, Fibroblast Activity and Antibacterial Properties of Poly(3-Hydroxybutyric Acid-Co-3-Hydroxyvaleric Acid) Grafted with Chitosan and Chitooligosaccharide after Immobilized with Hyaluronic Acid. Biomaterials.

[32] Van den Dolder J., de Ruijter A.J.E., Spauwen P.H.M., Jansen J.A. (2003). Observations on the Effect of BMP-2 on Rat Bone Marrow Cells Cultured on Titanium Substrates of Different Roughness. Biomaterials.

[33] Ji G.-Z., Wei X., Chen G.-Q. (2009). Growth of Human Umbilical Cord Wharton’s Jelly-Derived Mesenchymal Stem Cells on the Terpolyester Poly(3-Hydroxybutyrate-Co-3-Hydroxyvalerate-Co-3-Hydroxyhexanoate). J. Biomater. Sci. Polym. Ed..

[34] Gennes P.G. (1987). Smachivanie: Statika i Dinamika. Uspekhi Fiz. Nauk.

[35] Ko H.-F., Sfeir C., Kumta P.N. (2010). Novel Synthesis Strategies for Natural Polymer and Composite Biomaterials as Potential Scaffolds for Tissue Engineering. Philos. Trans. R. Soc. A Math. Phys. Eng. Sci..

[36] Kroeze R., Helder M., Govaert L., Smit T. (2009). Biodegradable Polymers in Bone Tissue Engineering. Materials.

[37] Chen G.-Q. (2010). Plastics Completely Synthesized by Bacteria: Polyhydroxyalkanoates. Plastics from Bacteria.

[38] Sudesh K., Abe H. (2010). Practical Guide to Microbial Polyhydroxyalkanoates.

[39] Laycock B., Halley P., Pratt S., Werker A., Lant P. (2013). The Chemomechanical Properties of Microbial Polyhydroxyalkanoates. Prog. Polym. Sci..

[40] Volova T.G., Shishatskaya E., Sinskey A.J. (2013). Degradable Polymers: Production, Properties, Applications.

[41] Chen G.-Q., Chen X.-Y., Wu F.-Q., Chen J.-C. (2020). Polyhydroxyalkanoates (PHA) toward Cost Competitiveness and Functionality. Adv. Ind. Eng. Polym. Res..

[42] Mitra R., Xu T., Chen G., Xiang H., Han J. (2022). An Updated Overview on the Regulatory Circuits of Polyhydroxyalkanoates Synthesis. Microb. Biotechnol..

[43] Tan D., Wang Y., Tong Y., Chen G.-Q. (2021). Grand Challenges for Industrializing Polyhydroxyalkanoates (PHAs). Trends Biotechnol..

[44] Koller M., Mukherjee A. (2022). A New Wave of Industrialization of PHA Biopolyesters. Bioengineering.

[45] Tarrahi R., Fathi Z., Seydibeyoğlu M.Ö., Doustkhah E., Khataee A. (2020). Polyhydroxyalkanoates (PHA): From Production to Nanoarchitecture. Int. J. Biol. Macromol..

[46] Kalia V.C. (2019). Biotechnological Applications of Polyhydroxyalkanoates.

[47] Popa M.S., Frone A.N., Panaitescu D.M. (2022). Polyhydroxybutyrate Blends: A Solution for Biodegradable Packaging?. Int. J. Biol. Macromol..

[48] Koller M., Mukherjee A. (2020). Polyhydroxyalkanoates—Linking Properties, Applications and End-of-Life Options. Chem. Biochem. Eng. Q..

[49] Polyhydroxyalkanoate (PHA) Market by Type (Short Chain Length, Medium Chain Length), Production Method (Sugar Fermentation, Vegetable Oil Fermentation), Application (Packaging & Food Services, Biomedical) and Region—Global Forecast to 2027. https://www.marketsandmarkets.com/Market-Reports/pha-market-395.html.

[50] Dalton B., Bhagabati P., de Micco J., Padamati R.B., O’Connor K. (2022). A Review on Biological Synthesis of the Biodegradable Polymers Polyhydroxyalkanoates and the Development of Multiple Applications. Catalysts.

[51] Palmeiro-Sánchez T., O’Flaherty V., Lens P.N.L. (2022). Polyhydroxyalkanoate Bio-Production and Its Rise as Biomaterial of the Future. J. Biotechnol..

[52] Adeleye A.T., Odoh C.K., Enudi O.C., Banjoko O.O., Osiboye O.O., Toluwalope Odediran E., Louis H. (2020). Sustainable Synthesis and Applications of Polyhydroxyalkanoates (PHAs) from Biomass. Process Biochem..

[53] Chen G.-Q., Wu Q. (2005). The Application of Polyhydroxyalkanoates as Tissue Engineering Materials. Biomaterials.

[54] Volova T.G., Vinnik Y.S., Shishatskaya E.I., Markelova N.M., Zaikov G.E. (2017). Natural-Based Polymers for Biomedical Applications.

[55] Guo W., Yang K., Qin X., Luo R., Wang H., Huang R. (2022). Polyhydroxyalkanoates in Tissue Repair and Regeneration. Eng. Regen..

[56] Singh A.K., Srivastava J.K., Chandel A.K., Sharma L., Mallick N., Singh S.P. (2019). Biomedical Applications of Microbially Engineered Polyhydroxyalkanoates: An Insight into Recent Advances, Bottlenecks, and Solutions. Appl. Microbiol. Biotechnol..

[57] Asare E., Gregory D.A., Fricker A., Marcello E., Paxinou A., Taylor C.S., Haycock J.W., Roy I. (2020). Polyhydroxyalkanoates, Their Processing and Biomedical Applications. The Handbook of Polyhydroxyalkanoates.

[58] Philip S., Keshavarz T., Roy I. (2007). Polyhydroxyalkanoates: Biodegradable Polymers with a Range of Applications. J. Chem. Technol. Biotechnol..

[59] Sharma V., Sehgal R., Gupta R. (2021). Polyhydroxyalkanoate (PHA): Properties and Modifications. Polymer.

[60] Singh M., Kumar P., Ray S., Kalia V.C. (2015). Challenges and Opportunities for Customizing Polyhydroxyalkanoates. Indian J. Microbiol..

[61] Li Z., Yang J., Loh X.J. (2016). Polyhydroxyalkanoates: Opening Doors for a Sustainable Future. NPG Asia Mater..

[62] Anjum A., Zuber M., Zia K.M., Noreen A., Anjum M.N., Tabasum S. (2016). Microbial Production of Polyhydroxyalkanoates (PHAs) and Its Copolymers: A Review of Recent Advancements. Int. J. Biol. Macromol..

[63] Riveiro A., Maçon A.L.B., del Val J., Comesaña R., Pou J. (2018). Laser Surface Texturing of Polymers for Biomedical Applications. Front. Phys..

[64] Slepicka P., Michaljanicova I., Svorcik V. (2013). Control.lled Biopolymer Roughness Induced by Plasma and Excimer Laser Treatment. Express Polym. Lett..

[65] Ortiz R., Aurrekoetxea-Rodríguez I., Rommel M., Quintana I., Vivanco M., Toca-Herrera J. (2018). Laser Surface Microstructuring of a Bio-Resorbable Polymer to Anchor Stem Cells, Control Adipocyte Morphology, and Promote Osteogenesis. Polymers.

[66] Volova T., Kiselev E., Nemtsev I., Lukyanenko A., Sukovatyi A., Kuzmin A., Ryltseva G., Shishatskaya E. (2021). Properties of Degradable Polyhydroxyalkanoates with Different Monomer Compositions. Int. J. Biol. Macromol..

[67] Braunegg G., Sonnleitner B., Lafferty R.M. (1978). A Rapid Gas Chromatographic Method for the Determination of Poly-?-Hydroxybutyric Acid in Microbial Biomass. Eur. J. Appl. Microbiol. Biotechnol..

[68] (1982). Surface Roughness—Parameters, Their Values and General Rules for Specifying Requirements.

[69] (2009). Medical Products. Estimating Biological Effects of Medical Products.

[70] (1985). International Guiding Principles for Biomedical Research Involving Animals.

[71] (2000). Declaration of Helsinki on Treating Research Animals with Respect.

[72] (1986). European Convention for the Protection of Vertebrate Animals Used for Experimental and Other Scientific Purposes.

[73] Surguchenko V.A., Ponomareva A.S., Efimov A.E., Nemets E.A., Agapov I., Sevastianov V. (2012). Characteristics of adhesion and proliferation of mouse NIH/3T3 fibroblasts on the poly(3-hydroxybutyrate-co-3-hydroxyvalerate) films with different surface roughness values. Vestn. Transpl. I Iskusstv. Organov.

[74] Bera P., Kotamreddy J.N.R., Samanta T., Maiti S., Mitra A. (2015). Inter-Specific Variation in Headspace Scent Volatiles Composition of Four Commercially Cultivated Jasmine Flowers. Nat. Prod. Res..

[75] Chanprateep S. (2010). Current Trends in Biodegradable Polyhydroxyalkanoates. J. Biosci. Bioeng..

[76] Zhang J., Shishatskaya E.I., Volova T.G., da Silva L.F., Chen G.-Q. (2018). Polyhydroxyalkanoates (PHA) for Therapeutic Applications. Mater. Sci. Eng. C.

[77] Wang Y.-W., Wu Q., Chen G.-Q. (2004). Attachment, Proliferation and Differentiation of Osteoblasts on Random Biopolyester Poly(3-Hydroxybutyrate-Co-3-Hydroxyhexanoate) Scaffolds. Biomaterials.

[78] Wang Y.-W., Yang F., Wu Q., Cheng Y., Yu P.H.F., Chen J., Chen G.-Q. (2005). Effect of Composition of Poly(3-Hydroxybutyrate-Co-3-Hydroxyhexanoate) on Growth of Fibroblast and Osteoblast. Biomaterials.

[79] Taylor G. (1969). Electrically Driven Jets. Proc. R. Soc. Lond. A Math. Phys. Sci..

[80] Volova T.G., Shishatskaya E.I., Gordeev S.A. (2006). Characterization of Ultrafine Fibers Electrostatically Spun from Poly(Hydroxybutyrate/Hydroxyvalerate) Solutions: Scientific Publication. Perspektivnye Materialy.

[81] Tong H.-W., Wang M. (2007). Electrospinning of Aligned Biodegradable Polymer Fibers and Composite Fibers for Tissue Engineering Applications. J. Nanosci. Nanotechnol..

[82] Tong H.-W., Wang M. (2011). Electrospinning of Poly(Hydroxybutyrate- *Co* -Hydroxyvalerate) Fibrous Scaffolds for Tissue Engineering Applications: Effects of Electrospinning Parameters and Solution Properties. J. Macromol. Sci. Part B.

[83] Volova T., Goncharov D., Sukovatyi A., Shabanov A., Nikolaeva E., Shishatskaya E. (2014). Electrospinning of Polyhydroxyalkanoate Fibrous Scaffolds: Effects on Electrospinning Parameters on Structure and Properties. J. Biomater. Sci. Polym. Ed..

[84] Wang Y., Gao R., Wang P.-P., Jian J., Jiang X.-L., Yan C., Lin X., Wu L., Chen G.-Q., Wu Q. (2012). The Differential Effects of Aligned Electrospun PHBHHx Fibers on Adipogenic and Osteogenic Potential of MSCs through the Regulation of PPARγ Signaling. Biomaterials.

[85] Yu B.-Y., Chen P.-Y., Sun Y.-M., Lee Y.-T., Young T.-H. (2012). Response of Human Mesenchymal Stem Cells (HMSCs) to the Topographic Variation of Poly(3-Hydroxybutyrate-Co-3-Hydroxyhexanoate) (PHBHHx) Films. J. Biomater. Sci. Polym. Ed..

[86] Wong S.-C., Baji A., Leng S. (2008). Effect of Fiber Diameter on Tensile Properties of Electrospun Poly(ε-Caprolactone). Polymer.

[87] Baji A., Mai Y.-W., Wong S.-C., Abtahi M., Chen P. (2010). Electrospinning of Polymer Nanofibers: Effects on Oriented Morphology, Structures and Tensile Properties. Compos. Sci. Technol..

[88] Baker S.C., Atkin N., Gunning P.A., Granville N., Wilson K., Wilson D., Southgate J. (2006). Characterisation of Electrospun Polystyrene Scaffolds for Three-Dimensional in Vitro Biological Studies. Biomaterials.

[89] Wang C., Chien H.-S., Yan K.-W., Hung C.-L., Hung K.-L., Tsai S.-J., Jhang H.-J. (2009). Correlation between Processing Parameters and Microstructure of Electrospun Poly(D,l-Lactic Acid) Nanofibers. Polymer.

[90] Li Q., Yang Y., Jia Z., Guan Z. (2007). Experimental Investigation of the Effect of Processing Parameters on the Formation of Electrospun Polyethylene Oxide Nanofibers. Gaodianya Jishu High Volt. Eng..

[91] Cheng M.-L., Lin C.-C., Su H.-L., Chen P.-Y., Sun Y.-M. (2008). Processing and Characterization of Electrospun Poly(3-Hydroxybutyrate-Co-3-Hydroxyhexanoate) Nanofibrous Membranes. Polymer.

[92] Yördem O.S., Papila M., Menceloğlu Y.Z. (2008). Effects of Electrospinning Parameters on Polyacrylonitrile Nanofiber Diameter: An Investigation by Response Surface Methodology. Mater. Des..

[93] Huang Z.-M., Zhang Y.-Z., Kotaki M., Ramakrishna S. (2003). A Review on Polymer Nanofibers by Electrospinning and Their Applications in Nanocomposites. Compos. Sci. Technol..

[94] Kenawy E.-R., Layman J.M., Watkins J.R., Bowlin G.L., Matthews J.A., Simpson D.G., Wnek G.E. (2003). Electrospinning of Poly(Ethylene-Co-Vinyl Alcohol) Fibers. Biomaterials.

[95] Kessick R., Fenn J., Tepper G. (2004). The Use of AC Potentials in Electrospraying and Electrospinning Processes. Polymer.

[96] Khil M.S., Kim H.Y., Kim M.S., Park S.Y., Lee D.-R. (2004). Nanofibrous Mats of Poly(Trimethylene Terephthalate) via Electrospinning. Polymer.

[97] Mo X.M., Xu C.Y., Kotaki M., Ramakrishna S. (2004). Electrospun P(LLA-CL) Nanofiber: A Biomimetic Extracellular Matrix for Smooth Muscle Cell and Endothelial Cell Proliferation. Biomaterials.

[98] Theron S.A., Zussman E., Yarin A.L. (2004). Experimental Investigation of the Governing Parameters in the Electrospinning of Polymer Solutions. Polymer.

[99] Riboldi S.A., Sampaolesi M., Neuenschwander P., Cossu G., Mantero S. (2005). Electrospun Degradable Polyesterurethane Membranes: Potential Scaffolds for Skeletal Muscle Tissue Engineering. Biomaterials.

[100] Sombatmankhong K., Sanchavanakit N., Pavasant P., Supaphol P. (2007). Bone Scaffolds from Electrospun Fiber Mats of Poly(3-Hydroxybutyrate), Poly(3-Hydroxybutyrate-Co-3-Hydroxyvalerate) and Their Blend. Polymer.

[101] Ying T.H., Ishii D., Mahara A., Murakami S., Yamaoka T., Sudesh K., Samian R., Fujita M., Maeda M., Iwata T. (2008). Scaffolds from Electrospun Polyhydroxyalkanoate Copolymers: Fabrication, Characterization, Bioabsorption and Tissue Response. Biomaterials.

[102] Ishii D., Ying T., Yamaoka T., Iwata T. (2009). Characterization and Biocompatibility of Biopolyester Nanofibers. Materials.

[103] Bashur C.A., Dahlgren L.A., Goldstein A.S. (2006). Effect of Fiber Diameter and Orientation on Fibroblast Morphology and Proliferation on Electrospun Poly(d,l-Lactic-Co-Glycolic Acid) Meshes. Biomaterials.

[104] Pham Q.P., Sharma U., Mikos A.G. (2006). Electrospinning of Polymeric Nanofibers for Tissue Engineering Applications: A Review. Tissue Eng..

[105] Sill T.J., von Recum H.A. (2008). Electrospinning: Applications in Drug Delivery and Tissue Engineering. Biomaterials.

[106] Lowery J.L., Datta N., Rutledge G.C. (2010). Effect of Fiber Diameter, Pore Size and Seeding Method on Growth of Human Dermal Fibroblasts in Electrospun Poly(ε-Caprolactone) Fibrous Mats. Biomaterials.

[107] Minchenko A. (2003). Wounds. Treatment and Prevention of Complications.

[108] Abayev Y.K. (2006). Surgeon Reference Book. Wounds and Wound Infection.

[109] Adamyan A.A., Dobysh S.v, Kilimchuk L.E., Goryunov S.v, Efimenko N.A., Shibanov E.A., Khrupkin V.I., Leshnevskii A.v, Emel’yanov A.v, Nuzhdin O.I., Fedorov V.D., Chizh I.M. (2000). Biologically Active Dressings in the Complex. Treatment of Purulent-Necrotic Wounds.

[110] FDA Clears First of Its Kind Suture Made Using DNA Technology. http://www.fda.gov/bbs/topics/NEWS/2007/NEW01560.html.

[111] Martin D.P., Skraly F.A., Williams S.F. (2005). Polyhydroxyalkanoate Compositions Having Controlled Degradation Rats. U.S. Patent.

[112] Shishatskaya E.I., Nikolaeva E.D., Vinogradova O.N., Volova T.G. (2016). Experimental Wound Dressings of Degradable PHA for Skin Defect Repair. J. Mater. Sci. Mater. Med..

[113] Volova T.G., Shumilova A.A., Nikolaeva E.D., Kirichenko A.K., Shishatskaya E.I. (2019). Biotechnological Wound Dressings Based on Bacterial Cellulose and Degradable Copolymer P(3HB/4HB). Int. J. Biol. Macromol..

[114] Guo W., Wang X., Yang C., Huang R., Wang H., Zhao Y. (2022). Microfluidic 3D Printing Polyhydroxyalkanoates-Based Bionic Skin for Wound Healing. Mater. Futures.

